# Accelerating cancer therapy review: a cross-sectional analysis of expedited approval in China, 2005–2021

**DOI:** 10.1186/s12885-026-15749-1

**Published:** 2026-02-16

**Authors:** Yun Tian, Xingyu Liu, Xingchen Liu, Xiaoyong Liu, Shuchen Hu, Xiaodong Liu, Caijun Yang, Yu Fang

**Affiliations:** 1 Department of Pharmacy Administration, School of Pharmacy, Xi’an Jiaotong University, No 76, Yanta West Road, Xi’an, Shaanxi China; 2 Center for Drug Safety and Policy Research, Xi’an Jiaotong University, Xi’an, Shaanxi China; 3 Department of Pharmacy, Shaanxi Provincial Cancer Hospital, Xi’an, Shaanxi China

**Keywords:** Cancer, Expedited approval, Pivotal clinical trials, China

## Abstract

**Background:**

China’s Expedited Approval (EA) pathways, launched in 2005, aim to accelerate patient access to novel cancer therapies. Yet the clinical value and regulatory efficiency of oncology drugs approved under EA have not been comprehensively evaluated in a Chinese context or compared against global benchmarks.

**Methods:**

We conducted a cross-sectional analysis of all malignant hematology and oncology drugs granted EA by China’s National Medical Products Administration between 2005 and 2021. Publicly available CDE reports, clinical trial registries, and literature sources were used to extract indication characteristics, review durations, trial design features, and ESMO-MCBS scores. Descriptive statistics summarized drug and trial features. Kruskal-Wallis tests compared median review times across factors; multiregional trial design and clinical benefit associations were explored qualitatively.

**Results:**

Ninety-nine drugs were granted expedited approval for 144 indications, 68% were small-molecule agents and 32% biologics; 86% underwent Priority Review, 28% Special Approval, and 24% Conditional Approval. Median review duration declined from over 1 000 days (2014&2016) to ~ 300 days post-2015. Imported drugs (median 289 days) were reviewed faster than domestic ones (401 days). Of 137 pivotal trials supporting new indications, 70% were randomized and 66% parallel-controlled; primary endpoints were ORR (43%) and PFS (37%). Among 86 solid-tumor trials with ESMO-MCBS data, 34% achieved scores indicating meaningful clinical benefit (grades 4–5 or A–B).

**Conclusion:**

China’s EA program has markedly accelerated oncology drug approval timelines to levels approaching the EMA, though still trailing the FDA. While most pivotal trials used surrogate endpoints, only one-third of solid-tumor indications demonstrated high clinical benefit per ESMO-MCBS. To balance expedited access with therapeutic value, future reforms should emphasize early benefit assessment, real-world outcomes, and alignment of surrogate endpoints with overall survival benefits.

**Supplementary Information:**

The online version contains supplementary material available at 10.1186/s12885-026-15749-1.

## Introduction

Cancer poses a significant societal, public health, and economic burden globally, accounting for nearly one in six deaths overall and one in four deaths from noncommunicable diseases [1]. China faces a substantial cancer burden, with a cancer spectrum similar to both developed and developing countries [2]. According to the Global Cancer Observatory (GLOBOCAN) 2020 database, China reports the highest incidence of new cancer cases (4.57 million) and cancer deaths (3 million), accounting for 23.7% and 30% of the global totals, respectively [3]. This underscores an urgent need for innovative therapies to provide sustained benefits to cancer patients.

Recent decades have witnessed remarkable advancements in oncology therapies, including immunotherapy, stem cell-based therapies, and nanocarrier-based therapies [4]. These accelerations of market authorization are largely attributed to the regulatory reforms that introduce expedited programs; such programs do not create new drugs, but rather hasten patient access to novel therapies by reducing review timelines or enabling earlier approval based on surrogate endpoints [5]. Between December 1992 and December 2021, the US Food and Drug Administration (FDA) approved 294 drugs under its accelerated approval program, with 61% for oncology (including hematology-oncology) indications [6]. Similarly, Chinese authorities approved 94 new anticancer drugs between January 2005 and May 2021, primarily through Expedited Approval (EA) programs [7]. Cancer is the indication with the highest proportion of expedited approval programs in both countries [8].

China’s EA program began in 2005 as the Special Review and Approval (commonly referred to as Special Approval, SA) program to address public health emergencies. In January 2009, the Special Review (SR) pathway was proposed to address the unmet medical needs for AIDS, malignant tumors, rare diseases, and other serious conditions, inspired by the FDA’s Priority Review and Fast Track programs. The SR pathway featured shortened review timelines, early communication opportunities, and rolling review processes but had minimal impact on overall New Drug Application (NDA) review and total development duration. In 2015, the Priority Review (PR) program was established to expedite the review timeframe and reduce the registration backlogs [9]. By 2020, the PR program had largely replaced the routine use of SA for non-emergency indications, due to functional overlap between the two routes. In December 2017, the Conditional Approval (CA) pathway was established to further shorten drug clinical trial development time for drugs demonstrating high clinical value over existing therapeutics for life-threatening or rare conditions, allowing for approval based on less comprehensive clinical data or surrogate endpoints that reasonably predict clinical benefits [10]. Notably, CA may be subject to withdrawal or indication revocation if confirmatory trials fail to verify the anticipated clinical benefit. Since 2020, China’s EA programs have aligned with those of the FDA and EMA, encompassing special approval, priority review, conditional approval and breakthrough therapy [11, 12].

The experience of the National Medical Products Administration (NMPA) with EA programs for oncology drugs was first documented in 2005 [13]. However, there are limited reports on how expedited review and approval initiatives, alongside the emergence of new therapeutic classes over the past decades, have influenced the speed of review and approval, as well as the clinical value of new cancer drugs approved through EA programs. This cross-sectional study comprehensively analyzes and reviews the EA approvals of malignant hematology and oncology drugs and biologics in China from 2005 to 2021, examining factors influencing review time, the characteristics of pivotal clinical trials, and the clinical benefit of these oncology drugs.

## Materials and methods

This cross-sectional study did not involve individual patient information; it exclusively utilized publicly available data from clinical trials and regulatory authorities, which had been previously reported. Consequently, accordance with 45 CFR§ 46, this study was deemed to not require institutional review board approval. This study followed the Strengthening the Reporting of Observational Studies in Epidemiology (STROBE) reporting guideline.

### Data sources and sample selection

To identify all cancer drugs approved under EA programs for malignant hematology and oncology indications, we examined the annual drug review reports and marketed drug database from the Center of Drug Evaluation (CDE). Our database inception in 2005 coincided with the release of CDE’s initial guideline on clinical trial results reporting, with relevant oncology drug literature on EA tracking back to the same year. The annual CDE review reports began to release the EA drug list in 2013. Therefore, to complement data prior to 2013, we referenced two additional papers [14, 15]. For each newly approved drug, we retrieved information on the first indication and all subsequent indications extensions up to December 31, 2021, including registration class (Abbreviated New Drug Application, New Drug Application, or Investigational New Drug), approval class (initial marketing or new indication supplement), sponsor nationality, cancer type, EA designation, and pivotal trial features. Public health insurance classification was obtained from the 2023 National Reimbursement Drug List (NRDL).

For trial information, we searched the official Chinese clinical trials website (chinadrugtrials.org.cn) and clinicaltrials.gov, including trial name, pivotal clinical trial number (NCT No.), designs, randomization, blinding, control groups, enrollment numbers and primary endpoints. When multiple endpoints supported approval, we selected the most clinically relevant one. The bridging studies conducted in China for imported drugs were also collected.

To address the limitations of publicly available data, we cross-checked our sample using the Pharnexcloud database by EA acceptance number, which contains new drug review information on investigational drugs. Extracted information included registration applicants, mechanism of action, EA review overview (new molecular entity, EA application date, approval date, review conclusion), and review duration (calculated from application receipt to marketing authorization in calendar days).

The clinical benefits of pivotal trials supporting EA for solid tumor drugs were evaluated using the European Society for Medical Oncology-Magnitude of Clinical Benefit Scale (ESMO-MCBS). The ESMO-MCBS grading highlights the treatment that substantially improve the duration of survival and/or the quality of life (QoL) of patients with cancer and aims to distinguish those from studies demonstrating only moderate, minor, or marginal clinical benefit. Scores were incorporated directly for therapies already graded by ESMO or assessed by our team according to ESMO-MCBS criteria [16]. For indications supported by both global and bridging trials, international clinical trial results determined the score.

### Statistical analysis

Descriptive statistics characterized indication features and clinical trial data. Numerical data were described using medians and interquartile ranges. The Kruskal-Wallis H test assessed approval speed variations among factors. Chi-square test and rank-sum test analyzed categorical and continuous variables, respectively. Data were organized with Excel 2019 and analyzed using IBM SPSS 26. Significance was set at *P* < 0.05.

## Results

### EAs of oncology drugs: indication characteristics

From 2005 to 2021, the NMPA granted EA to 99 malignant hematology and oncology drugs for 144 indications, based on 144 trials and 19 bioequivalence studies (Table 1 and eTable S1). Among the 144 indications, there were more new molecular entities (98, 68.1%) than novel biologics (46, 31.9%). More indications underwent priority review (124, 86.1%), followed by receiving special approval (41, 28.5%) and conditional approval (35, 24.3%). These expedited pathways are not mutually exclusive, with 57 indications reviewed through two or three pathways.

**Table 1:** Characteristics of 144 indications receiving EA from NMPA, 2005–2021

Indication characteristics	Types	Indications^a^ No (%)	Median review time^b^ (days)	*P* value
Drug class	New molecular entity	98(68.1%)	352	**0.031**
	Novel biologic	46(31.9%)	293.5	
Expedited program ^c^	Special Approval	41(28.5%)	361	0.809^d^
	Priority Review	124(86.1%)	334	
	Conditional Approval	35(24.3%)	292	
	Breakthrough Therapy	4(2.8%)	271	
Number of Expedited program pathways	Single	87(60.4%)	334	0.362
	Dural	55(38.2%)	336	
	Three	2(1.4%)	251	
Reasons for EA inclusion	New drugs with significant clinical value	113(78.5%)	313.5	**0.044**
	Urgently needed overseas new drugs	8(5.6%)	264	
	National Science and Technology Major Project	4(2.8%)	657	
	Generic	19(13.2%)	571	
Review type	Abbreviated New Drug Application	19(13.2%)	571	**0.008**
	New Drug Application	121(84.0%)	309	
	Investigational New Drug	1(0.7%)	233	
	Clinical Trial Authorization	3(2.1%)	630	
Manufacturer location	Domestic	87(60.4%)	401	**0.006**
	Imported	57(39.6%)	289	
Approval Type	Novel	93(64.6%)	350	**<0.01**
	Supplemental	32(22.2%)	275.5	
	Generic	19(13.2%)	571	
Initial indications	Hematological malignancies	38(26.4%)	476	**0.007**
	Solid cancer*	106(73.6%)	301	
Mechanism of action	Tyrosine kinase inhibitor	55(38.2%)	329	**0.014**
	Monoclonal antibody	42(29.2%)	286	
	Cytotoxic drug	20(13.9%)	519.5	
	Endocrine therapy	9(6.3%)	354	
	Others*	18(12.5%)	384	
Medical insurance catalog	Yes*	115(79.9%)	349	0.199
	No	29(20.1%)	286	
Approved for marketing	Yes	140(97.2%)	331	0.476
	No	4(2.8%)	631.5	

Bold values indicate statistical significance (*p* < 0.05)

a: The sample size for the “indications” was 144

b: The sample size for “review time” was 142 because two unapproved drugs did not have review time

c: There were several medicines using multiple expedited program pathways, hence the total items were 204

d: In exploring the impact of expedited program, the confounding effects of multiple modalities in combination were excluded, and only drugs with single expedited program pathway were selected

*: the detailed classification is described in Table S1

The primary rationale for EA designation was clinical significance, focusing more on novel therapeutic agents (113, 78.5%), with additional consideration for urgent needs for overseas new drugs and generics during the policy’s initial stage. Domestic pharmaceutical companies originated 87 indications (60.4%), with the remainder from foreign manufacturers, predominantly from Europe and the United States. In terms of review type, 121 indications were approved via New Drug Application (NDA) and 19 underwent Abbreviated New Drug Application (ANDA) review for potential generic product approval.

EA indications primarily targeted solid tumors (73.6%), with a focus on thoracic malignancies, breast cancer, gastrointestinal cancer, and genitourinary cancers (eTable S2). By mechanism of action, more indications involved tyrosine kinase inhibitors (38.2%), followed by monoclonal antibodies (29.2%), and 19 indications involved other mechanisms (eTable S2). PD-1/PD-L1-targeting drugs represent the largest share (25, 45.5%) among monoclonal antibodies, followed by VEGF/VEGFR-targeting drugs (12, 21.8%) (eTable S2). Most approved drugs were indicated for a single specific use, whereas 10 medicines had broader applications encompassing multiple indications. Notably, camrelizumab received EA for 5 indications, followed by tislelizumab and nivolumab, each approved for 4 indications. Additionally, two generic drugs, pemetrexed and sorafenib, were included in priority review three times. Four drugs, including one generic drug, were not approved at the end.

Approximately 79.9% of these EA approved oncology drugs were included in the 2023 National Reimbursement Drug List (NRDL), with 50 drugs listed in the National Health Insurance and Price Negotiation List (eTable S2), significantly enhancing patient accessibility. Only 18 drugs required fully out-of-pocket payments or commercial medical insurance.

### Review time for EA and influencing factors

Details of review time for each drug are provided in eTable S3. The median time to approval varied by drug class, with new molecular entities (352 days) longer than novel biologics (293.5 days). Imported drugs had a shorter review time compared to domestic drugs, and similarly, drugs designated as urgently needed overseas—that is, medicines already approved and clinically validated abroad but identified as addressing unmet medical needs in China—also underwent shorter review timelines. Additionally, novel indications took longer review durations than supplemental indications. Solid cancer approvals averaged 301 days, whereas hematological malignancies took 476 days. Specifically, monoclonal antibody exhibited the shortest review time, whereas cytotoxic drugs had the longest. Comparing medicines included in the medical insurance catalog, the median review time was shortest for medicines that were included in both Class B national medical insurance drug directory and the national reimbursement negotiation drug list (eTable S2). In total, there were 19 drugs approved in less than 200 days, and 7 drugs took more than 1000 days (eTable S4).

### Trial characteristics and verification of benefit

We identified 137 pivotal clinical trials for 121 new indications (excluding 4 disapproval indications) and 20 bioavailability studies for 19 generics (Table 2, eTable S5 & S6). Among these new indications, more were supported by randomized (70.1%), parallel control (66.4%) and open (65.7%) trials. Most studies were phase 3 trials, with phase 2 studies comprising 35.8% and phase 1 clinical trials accounting for 2.9%. The most frequently reported clinical endpoints were overall response rate (ORR, 43.1%) and progression-free survival (PFS, 36.5%). In terms of clinical trials, international multicenter clinical trial (IMCT) and multi-center clinical trial (MCCT) are the two most common types. Among the 86 trials scored by the ESMO-MCBS, 29 (33.7%) demonstrated clinically meaningful benefit, while 57 trials fell below the ESMO-MCBS criteria (≤ 3), with 46 receiving a grade of 3 points (Table 2). In the 137 pivotal trials, the median enrollment was 303 participants (IQR: 113–501), with 17.5% of trials having small enrollments, less than 97 participants (eTable S7 & eFigure1).

**Table 2:** Characteristics of 137 pivotal trials supported for EA of new oncology drugs (excluding generics), 2005–2021

Indication characteristics	Types	Indications No (%)
Randomization	Non-randomized	41(29.9%)
	Randomized	96(70.1%)
Control	Single-arm	46(33.6%)
	Parallel	91(66.4%)
Blinding	Open	90(65.7%)
	Double-blinded	47(34.3%)
Trials Phase	Ⅰ	4(2.9%)
	Ⅱ	49(35.8%)
	Ⅲ	84(61.3%)
Primary endpoint	Overall Survival (OS)	28(20.4%)
	Progression-Free Survival (PFS)	50(36.5%)
	Overall Response Rate (ORR)	59(43.1%)
Types of clinical trials	International Multicenter Clinical Trial (IMCT)	71(51.8%)
	Bridging study	14(9.7%)
	China Joining Multi-Regional Clinical Trials(CJMRCT)	32(22.9%)
	Multi-Center Clinical Trial (MCCT)	64(46.7%)
	Single Center Clinical Trial (SCCT)	2(1.5%)
MCBS score for solid tumors*	Score 1	3(3.5%)
	Score 2	8(9.3%)
	Score 3	46(53.5%)
	Score 4	23(26.7%)
	Score 5/A	6(7.0%)

*: Regarding the clinical benefit of new drugs for solid tumors, 86 pivotal trials supporting approval were evaluable by the ESMO-MCBS. Of these, 10 trials lacked publicly available scores and were independently assessed using the MCBS scorecard. Therefore, there were only 86 indications with Magnitude of Clinical Benefit Scale (MCBS) score

### EAs of oncology drugs: annual trend changes

**Fig. 1:**
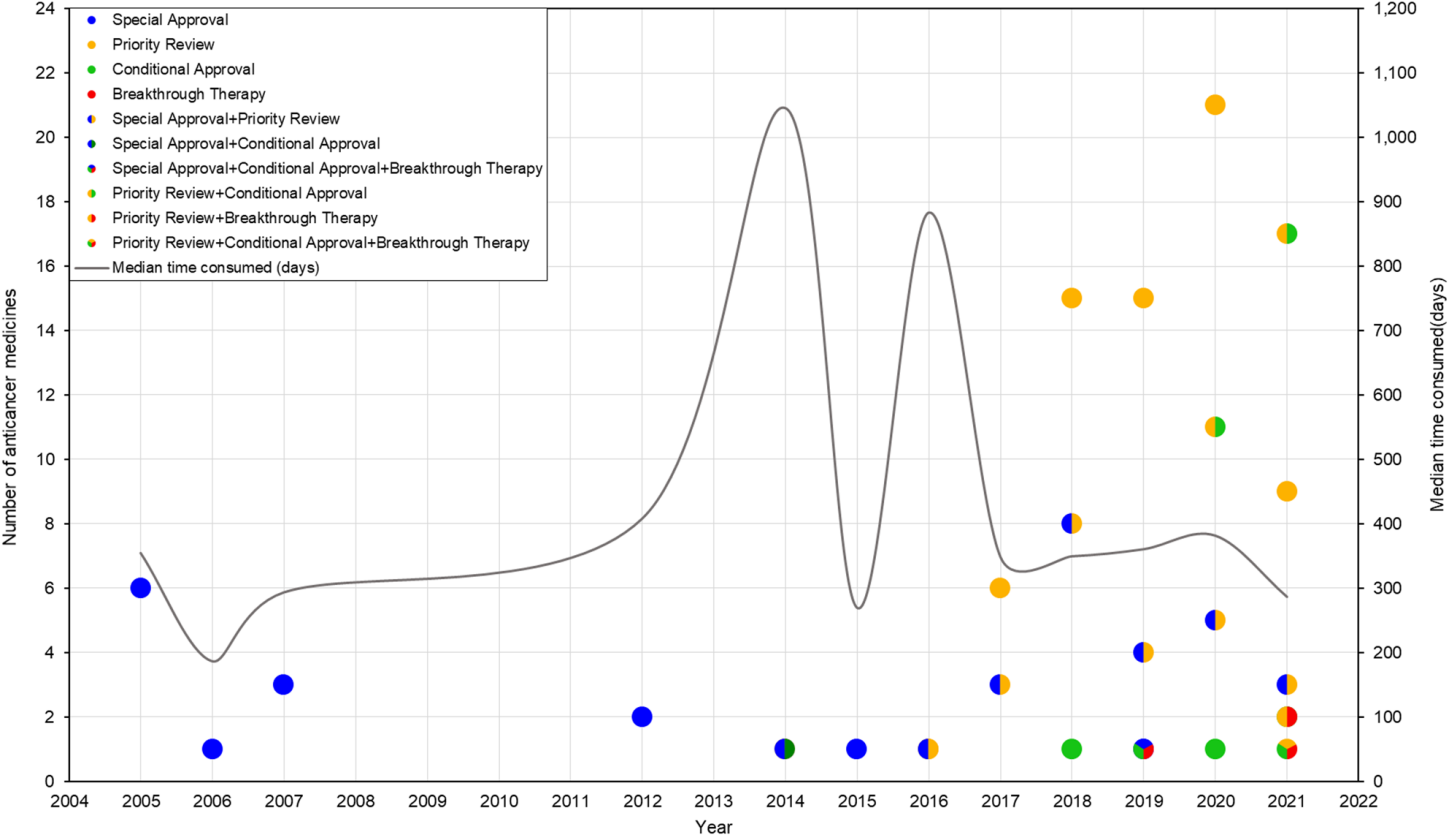
The approvals by year and pathway for anticancer medicines through EA

From 2005 to 2013, the median review time for oncology drugs under EA pathways exhibited an upward trend, culminating at over 1,000 days in 2014 and 2016. Subsequently, review time decreased markedly in 2015 and have since demonstrated a declining trend, stabilizing at approximately 300 days (Fig. 1).

**Fig. 2:**
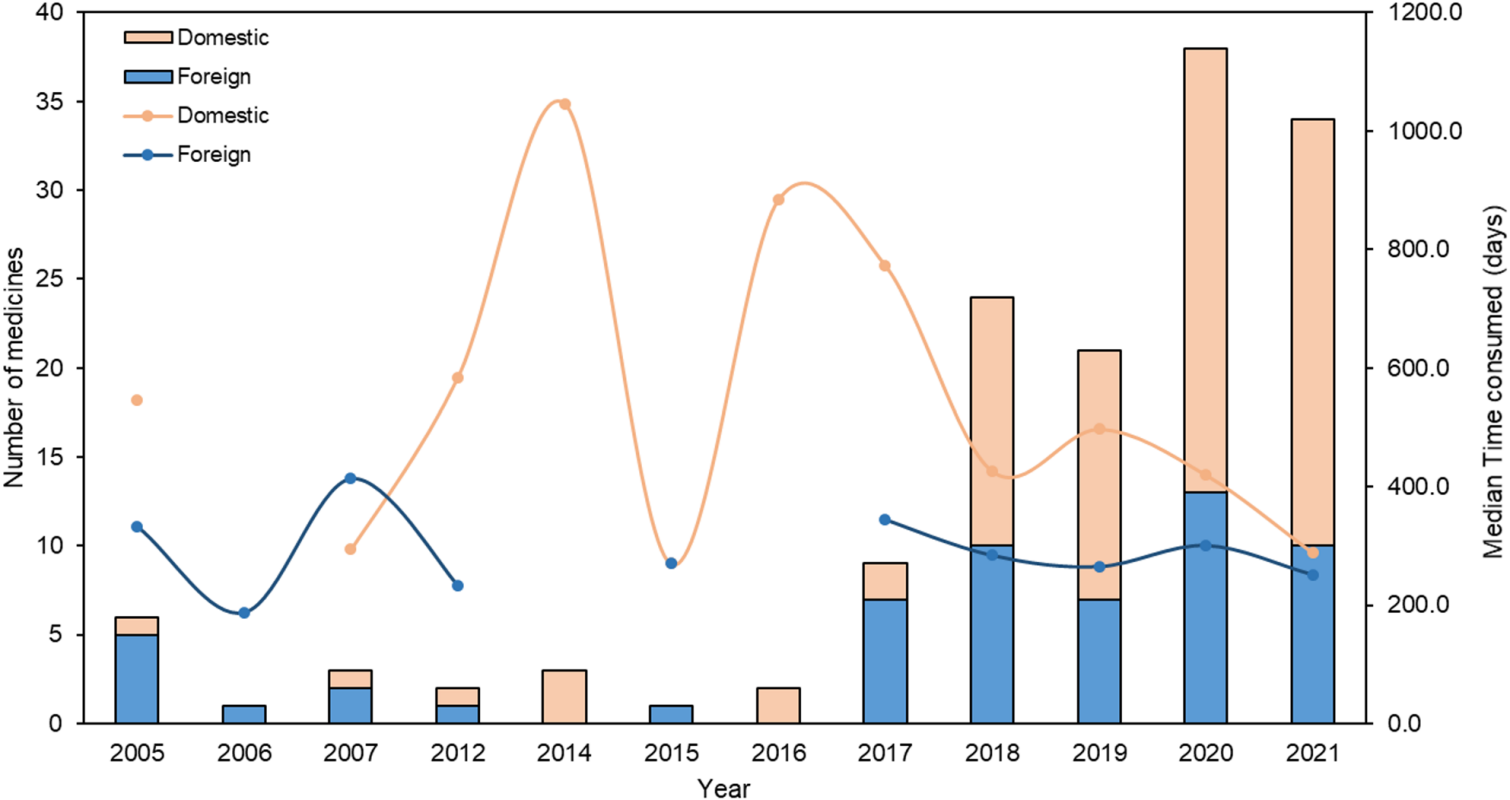
Number of medicines prioritized for review and number of review days by year

**Fig. 3:**
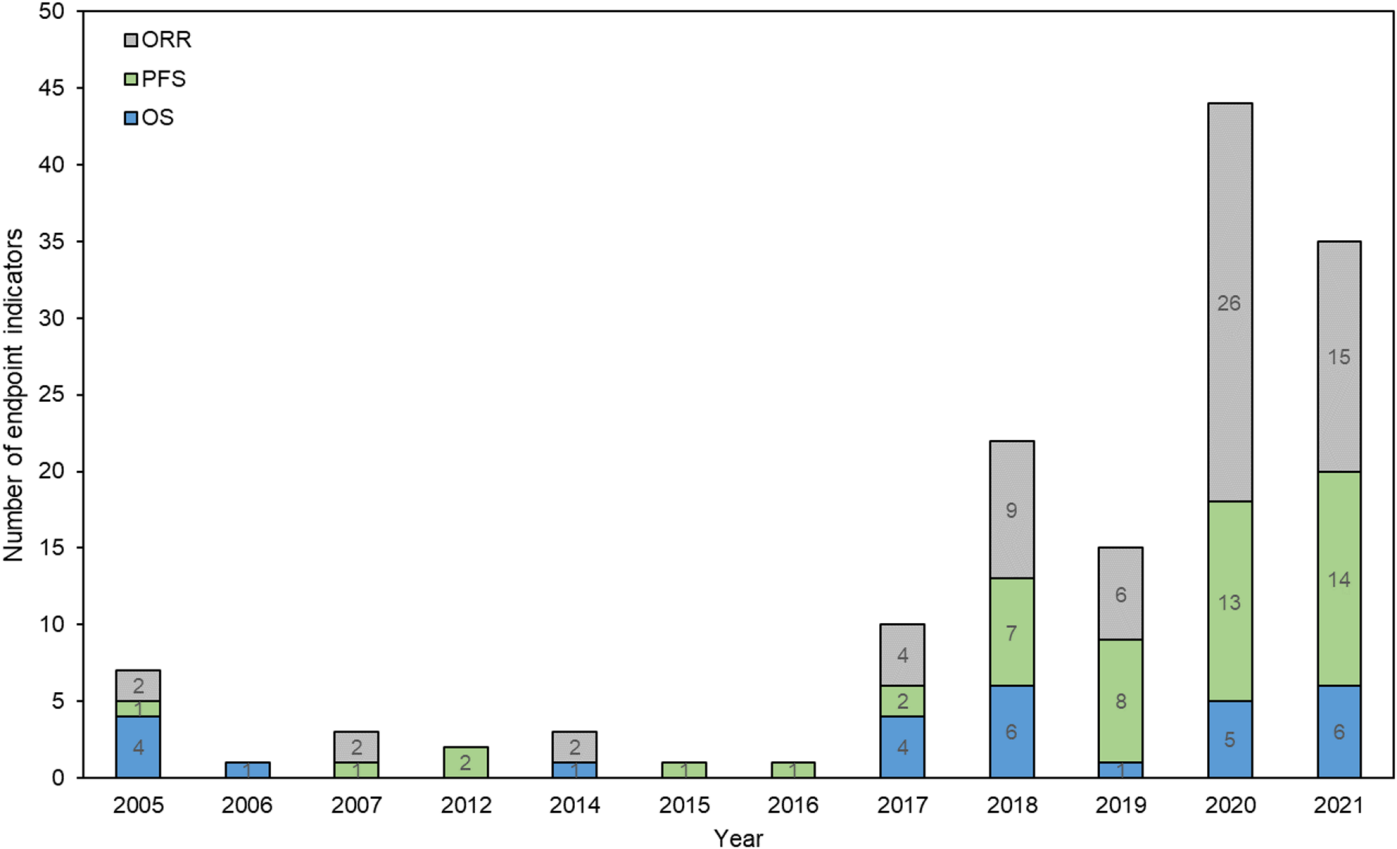
The primary endpoint indicators used in clinical trials

Initially, the number of domestically and internationally developed oncology drugs approved was low, but this figure gradually increased over time. The review timelines for both domestic and foreign drugs displayed fluctuations followed by a downward trend, with foreign drugs consistently requiring less time compared to domestic counterparts (Fig. 2). The overall response rate (ORR) was the primary endpoint in most years, particularly post-2017. Additionally, progression-free survival (PFS) was adopted frequently and exhibited a notable surge in 2020 (Fig. 3).

## Discussion

Over the past decade, China’s expedited approval (EA) policies have undergone continuous evolution and refinement, accompanied by revised drug registration regulations to accelerate access to innovative therapies, including treatments for severe diseases and rare disorders. Overall, the EA system is generally recognized to have evolved across four phases: the preliminary exploration stage (before November 2015), the backlog clearance stage (November 2015–December 2017), the reform and framework consolidation stage (December 2017–July 2020), and the innovation-driven optimization stage (July 2020–present).The focus of policy implementation has gradually shifted from resolving historical review delays to establishing a structured multi-pathway system, and further toward encouraging innovation and improving access efficiency for high-clinical-value therapies.

Among accelerated approvals in oncology, priority review remains the most frequently utilized pathway (124, 86.1%), followed by special approval (41, 28.5%) and conditional approval (35, 24.3%). Although routine approval is still common in oncology, these data indicate that a substantial proportion of cancer drugs seeking accelerated pathways predominantly rely on priority review. Drugs included in the National Science and Technology Major Project or on the Urgently Needed Overseas Drugs (UNOD) list are eligible to apply for priority review or special approval due to the absence of a separate fast-track route specific to them. UNOD drugs—typically with recognized clinical benefit internationally—may obtain EA approval and market authorization based on limited domestic clinical data, provided that no significant ethnic differences are expected [17].

Unlike the FDA or the EMA, China’s EA program not only prioritizes innovative oncology drugs but also favors high-clinical-value generic products, driven by unmet clinical needs and medication accessibility. Prior to 2020, fourteen generic cancer drugs, including anastrozole, pemetrexed and paclitaxel (albumin-bound), among others, were incorporated into the PR program based on bioequivalence data. These drugs serve as cornerstones of small-molecule cancer therapies. Pemetrexed, the top-selling chemotherapeutic drug globally since its US market introduction in 2004, saw its first generic version launched domestically in 2006. Albumin-bound paclitaxel offers advantages over paclitaxel and its liposomal formulation, and is preferred for preoperative neoadjuvant and late-stage palliative breast cancer therapy due to reduced allergic reactions. To enhance the accessibility and availability of high-clinical-value small-molecule drugs not domestically manufactured, the EA program continues to prioritize generic drugs to address unmet needs.

### Review time and possible influencing factors

China has witnessed significant improvements in drug review speed. The median review timeframe for cancer therapy approved through the EA program was 333.5 days. This is consistent with previous study where new anticancer drug applications decreased from 561 days (18.7 months) in 2008–2010 to 375 days (12.5 months) in January 2018-May 2021 in China [18]. In contrast, the FDA and EMA had median review durations of 200 days and 426 days, respectively, for oncology drugs [19], indicating that China’s review speed is nearly paralleling the EMA’s pace but still lags behind the FDA. The 2023 Annual Drug Review Report noted a reduction in the review timeframe for priority review and approval procedures, with the limit shortened from 200 days to 120 days for regular procedures. Specifically, the timeframe for reviewing clinically urgent drugs for rare diseases already marketed internationally was reduced to 70 days [10].

Among the 19 antineoplastic drugs reviewed in less than 200 days, those granted Priority Review were often supported by pivotal clinical trials demonstrating substantial clinical benefit. For example, Osimertinib had one of the shortest review durations (approximately 7 weeks) — it was granted approval in China in March 2017 shortly after its global approval, reflecting expedited regulatory processing under the PR pathway. Its underlying data (59% overall response rate among 411 T790M-positive NSCLC patients) indicated strong efficacy in a biomarker-defined population, which meets high unmet medical need and thus triggered priority review [20]. This supports the notion that therapies with clear molecular targets, robust efficacy signals, and biomarker-guided indications are more likely to benefit from accelerated approval pathways, thereby shortening regulatory review time.

Conversely, some EA program drugs experienced longer review timeframe than regular approvals, particularly those under Priority Review or Special Approval. Utidron, (utidelone) the first epothilone-based antitumor drug independently developed in China, had one of the longest review durations of 1,074 days after 2018. Its mechanism of action, while similar to paclitaxel, differs in binding site, thereby overcoming drug resistance issues and providing a new effective therapeutic option for advanced breast cancer patients resistant to paclitaxel. Based on the impressive data from gastrointestinal oncology study [21], utidron received Orphan Drug Designation from the FDA for gastric cancer treatment and was included in China’s National Reimbursement Drug List (NRDL) in 2023. Its prolonged review period may be associated with difficulty in eligible patients’ enrollments.

Factors that may have an impact on the review period are complex, however, minimal variability exists in the review timeframe for novel medications exhibiting significant clinical value or those imported to address urgent clinical needs.

### Domestic approvals of anticancer drugs

Prior to 2010, the majority of anticancer drugs approved in China were cytotoxic chemotherapeutic agents, predominantly generics. However, over the past two decades, a notable decline in cytotoxic drugs has been observed, accompanied by a rapid surge in novel targeted therapies and biosimilars. Our study reveals that over half of the drugs (60.42%, 87/144) were entirely developed by Chinese pharmaceutical companies. In the past five years, the number of applications from Chinese companies has surpassed those from foreign entities [22]. However, Chinese pharmaceutical companies still have a long way to go to achieve “true innovation”. Between 2005 and 2021, three-quarters of cancer drugs developed by Chinese pharmaceutical companies were not innovative in terms of either mechanism of action or therapy.

Four medications (Abivertinib, Geptanolimab, Dicycloplatin, and the generic Bortezomib) developed by domestic pharmaceutical companies were not approved by the NMPA of China. The primary rationale for the rejection of Abivertinib and Geptanolimab was the inability to demonstrate clear clinical benefit. In addition, Geptanolimab was denied because several anti-PD-(L)1 antibodies with highly similar mechanisms were already available on the market, resulting in therapeutic redundancy rather than added clinical value. Several immune checkpoint inhibitors that received EA based on surrogate endpoints have been withdrawn from their approved indications [23]. Since 2021, the pace of domestic PD-1 drug development and approval has slowed down—a trend driven by China’s regulatory focus on clinical value differentiation, particularly the NMPA’s guidance to avoid redundant R&D in highly saturated therapeutic areas [24].The generic formulation of Bortezomib was not approved due to multiple pharmaceutical manufactures submitting the same generic drug simultaneously. Dicycloplatin was not marketed due to other commercial reasons. Overall, four cancer drugs were terminated at the approval stage.

### Clinical trials

The development and registration strategy for imported drugs in China has evolved from conducting overseas post-approval bridging studies to engaging in pre-approval multiregional clinical trials (MRCTs) on a global scale over the past decade. Between 2008 and 2013, multinational pharmaceutical companies primarily introduced globally approved drugs into China through bridging studies, shaping China’s drug registration strategy. When a drug’s overall survival (OS) benefit is established in large-scale controlled trials, surrogate endpoints such as progression-free survival (PFS) and event-free survival (EFS) may be selected in bridging studies to enhance research efficiency. The guidelines issued by the NMPA in 2018 officially incorporated clinical data from overseas trials into the regulatory framework. Conditional approval granted based on overseas clinical trial data can be transitioned to regular approval upon completion of the requisite bridging trials [25].

The parallel advancement of global research and development is progressively gaining momentum. Zanubrutinib, the first domestically innovative drug to undergone global simultaneous development in its early stage, received dual approval from both FDA and NMPA, with pivotal clinical trials data mainly coming from Chinese patients. Drugs that undergo MRCTs can help to narrow the gap in drug availability between China and the United States, but they may also lead to a subsequent downgrading in the design of clinical endpoints in Chinese population.

As patients with tumors are now surviving longer with the availability of cutting-edge treatments, surrogate endpoints associated with survival benefit can be used to expedite the clinical trial process and facilitate early drug market entry, thereby addressing unmet medical needs. Composite outcome measures, such as PFS and disease-free survival (DFS), are increasingly used as surrogate endpoints in oncology research, frequently serving as the primary endpoint in pivotal trials that form the basis for FDA and EMA approvals [26]. In our study, 50 pivotal clinical trials (36.5%) selected PFS, DFS, EFS, or recurrence-free survival (RFS) as the surrogate endpoint. A retrospective study of 107 oncology drugs with 188 approved indications revealed that using PFS as a surrogate endpoint in registration trials shortened the mean time to obtain approval-supporting data by 11 months compared to using OS as the primary endpoint [27].

Our data also confirm that ORR is a prevalent endpoint in single-arm studies supporting the EA of anticancer therapies. An increasing number of cancer drug approvals in China rely on single-arm trials or preliminary survival data. A recent study revealed that, from 2000 to 2020, 82% of novel cancer drug approvals by the FDA were based on single clinical trial results [28]. In oncology, single-arm trial designs and response rate endpoints (with duration of response as supportive evidence) are prevalent because response rate is a reliable indicator of drug activity in malignant tumors that do not naturally regress. Furthermore, response rates can be interpreted within the context of single-arm trials for monotherapy drug regimens. For instance, Chidamide was approved in 2014 for relapsed and refractory peripheral T-cell lymphomas (PTCL) based primarily on a pivotal, multi-center, single-arm phase II clinical trial with ORR as the primary endpoint [29]. However, using single-arm trials to support EA has limitations, including small safety datasets, low-magnitude response rates that may not reliably predict clinical benefit, and the inability to establish differential contribution of effect for combination regimens. The FDA issued a draft guidance document aimed at improving oncology clinical trials for AA, stating that single-arm trials should only be conducted when RCTs are infeasible, given that low-magnitude response rates generally do not predict clinical benefit with reasonable certainty [30]. In 2021, the CDE released guidelines for the clinical value-based development of oncology drugs [24], clearly stating that the ultimate objective of new drug development should be to provide patients with superior treatment options, encompassing enhanced efficacy, safety, and convenience. In principle, single-arm trials are appropriate for the treatment of refractory or rare diseases that are severely life-threatening and lacking effective standard therapies, as well as for monotherapy demonstrating outstanding efficacy in early exploratory studies.

Composite endpoints are frequently used in clinical outcome trials to provide more endpoints, thereby increasing statistical power. However, the existence of qualitative heterogeneity of these endpoints makes interpreting trial results extremely challenging [31]. Furthermore, it is imperative to acknowledge that different surrogate endpoints exhibit unique functional attributes, necessitating a thorough analysis of potential bias inherent in composite outcomes. Notably, the selection of trial endpoints may vary across clinical trials targeting different stages of tumor diseases. In recognition of this, the NMPA has issued specific guidelines, such as the Technical Guidelines for Clinical Trial Endpoints for the approval of Advanced Non-Small Cell Lung Cancer [32], Advanced Hepatocellular Carcinoma [33], and Advanced Prostate Cancer [34], which outline considerations for the use of surrogate endpoints in various stages of diverse advanced tumors. Additionally, the transparent reporting of the component events is paramount in clinical studies to ensure consistent interpretation of clinical significance and enhance the reliability of oncology studies [35]. Even when a validated surrogate endpoint is adopted as the primary endpoint, continuous attention and follow-up on patients’ long-term survival data are imperative. In January 2022, the Guiding Principles for the Application of Patient-Reported Endpoints in Clinical Research and Development of Drugs (for Trial Implementation) was formally released [36], highlighting the importance of patient-reported outcomes in guiding drug registration-oriented clinical research.

### MCBS scores and clinical benefits

Although expedited pathways facilitate clinical development and regulatory review, they must be carefully balanced against the risk of market entry for drugs with limited clinical efficacy [37]. The WHO Expert Committee has endorsed the Cancer Medicine Working Group’s (CMWG) recommendation to use ESMO-MCBS as a screening tool to identify high-value cancer drugs for inclusion in the WHO Essential Medicines List (EML). To qualify for inclusion in the WHO EML, solid tumor treatments must achieve an ESMO-MCBS score of A or B in the curative setting or 4 or 5 in non-curative setting, indicating significant benefit. Conversely, drugs with scores of C or 1–3 are deemed low-benefit and ineligible.

Our analysis indicates that use of the EA program has increased over time, with clinical benefit verified in only 33.7% of the approved indications. From 2007 to 2021, 38.9% and 37.5% of new drug indications approved through the accelerated approval or conditional marketing authorization pathways in the US and Europe, respectively, demonstrated high therapeutic value [38]. Existing evidence suggests that anticancer drugs with higher ESMO-MCBS scores are more likely to transition from expedited to regular approval [39]. In our study, there were 57 trials scored 1 to 3 points using ESMO-MCBS version 1.1. Whether these trials represent low clinical benefit depends on specific context, such as the availability of subsequent treatment options, and should be evaluated on a case-by-case basis. Camrelizumab for esophageal cancer, Nivolumab for gastric cancer, and Disitamab Vedotin for metastatic gastric cancer scored 1 point. The first two have received regular approval and the third (self-developed drug targeting HER-2 ADC) is undergoing confirmatory trials. Treatment effects are heterogeneous, and some patients may derive substantial benefit from drugs with low ESMO-MCBS scores [40]. For oncology drugs deemed to have limited clinical benefit by ESMO-MCBS or other regulatory evaluations, potential benefits in biomarker-defined subpopulations should be further assessed, for example through stratification by predictive biomarkers such as HER2 status or PD-L1 combined positive score (CPS), as well as through patient-reported outcomes in real-world settings.

### Limitations

This study has several limitations. Firstly, we only considered official review times and did not account for pre-submission inquires or real-time oncology reviews employed by regulatory bodies, as this information is typically confidential and not accessible to the public. Secondly, cancer drugs approved for hematological malignancies lack ESMO-MCBS scores, which may potentially lead to an underestimation of the proportion of novel therapies that offer clinically meaningful benefits. Thirdly, when evaluating indications supported by multiple pivotal clinical trials, we prioritized global trial data over domestic trials when both were available, which may potentially overlook any discrepancies between international and domestic outcomes.

## Conclusion

Over a 17-year period, the Expedited Approval (EA) program has demonstrated its effectiveness in accelerating the approval of cancer therapies. These novel therapies are transforming cancer treatment landscapes in China by providing patients a richer variety of treatment options. Our cross-sectional study revealed that China’s review speed for new oncology therapies nearly parallels that of the EMA, yet still lags behind the FDA. Only approximately 20% of new cancer therapies approved in China between 2005 and 2021 had documented OS data. Moreover, verified clinical benefits were demonstrated in up to one-third of the indications. Given these findings, it is imperative for regulatory authorities and non-profit professional academic societies to develop more precise and instructive evaluation tools for value assessment to ensure the efficacy, safety, and public confidence in cancer medicines.

## Supplementary Information

Supplementary Material 1.

Supplementary Material 2.

## Data Availability

All data generated or analysed during this study are included in the supplementary information files.

## Abbreviations

AA: Accelerated Approval
ANDA: Abbreviated New Drug Application
CA: Conditional Approval
CDE: Center for Drug Evaluation
CMA: Conditional Marketing Authorization
DFS: Disease-Free Survival
EA: Expedited Approval
EMA: European Medicines Agency
EFS: Event-Free Survival
ESMO-MCBS: European Society for Medical Oncology–Magnitude of Clinical Benefit Scale
FDA: U.S. Food and Drug Administration
IMCT: International Multicenter Clinical Trial
OoL: Quality of Life
RCT: Randomized Controlled Trial
RFS: Recurrence-Free Survival
UNOD: Urgently Needed Overseas Drugs
MCCT: Multicenter Clinical Trial
MRCT: Multiregional Clinical Trial
NDA: New Drug Application
NMPA: National Medical Products Administration
NRDL: National Reimbursement Drug List
ORR: Objective Response Rate
OS: Overall Survival
PFS: Progression-Free Survival
PR: Priority Review
PRO: Patient-Reported Outcome
SR: Special Review
SA: Special Approval
TKI: Tyrosine Kinase Inhibitor
WHO: World Health Organization
