# Supplementary material for: Accelerating cancer therapy review: a cross-sectional analysis of expedited approval in China, 2005–2021
Source: BMC Cancer. 2026 Feb 16;26:390. doi: 10.1186/s12885-026-15749-1 (PMC13015170; doi:10.1186/s12885-026-15749-1)

## eTable S1: Expedited programs for oncology drugs from 2005-2021

| **Drug Name** | **Handling No.** | **Expedited programs** | **Expedited programs Amount** | **Reasons for inclusion** | **EP approval year** |
| --- | --- | --- | --- | --- | --- |
| Abemaciclib | JXHS1900144 | PR | 1 | New drugs with significant clinical value | 2020 |
| Abiraterone | JXHS1800011 | PR | 1 | New drugs with significant clinical value | 2018 |
| Avapritinib | JXHS2000050 | CA,PR | 2 | New drugs with significant clinical value | 2020 |
| Afatinib | JXHS1600009 | SAP,PR | 2 | New drugs with significant clinical value | 2017 |
| Axicabtagene ciloleucel | CXSS2000006 | CA,PR | 2 | New drugs with significant clinical value | 2020 |
| Alectinib | JXHS1800001 | PR | 1 | New drugs with significant clinical value | 2018 |
| Almonertinib | CXHS1900011 | SAP,PR,BT | 3 | New drugs with significant clinical value | 2019 |
| Almonertinib | CXHS2101017 | PR,BT | 2 | New drugs with significant clinical value | 2021 |
| Apalutamide | JXHS1900036 | PR | 1 | Urgently needed overseas new drugs | 2019 |
| Apalutamide | JXHS1900156 | PR | 1 | Urgently needed overseas new drugs | 2020 |
| Apatinib | CXHS1100133 | SAP | 1 | National Science and Technology Major Project | 2014 |
| Atezolizumab | JXSS2000002 | PR | 1 | New drugs with significant clinical value | 2020 |
| Atezolizumab | JXSS2000033 | CA,PR | 2 | New drugs with significant clinical value | 2021 |
| Axitinib | JXHS1400080 | SAP | 1 | Urgently needed overseas new drugs | 2015 |
| Icotinib | CXHL0502177 | SAP | 1 | New drugs with significant clinical value | 2007 |
| Icotinib | CXHS2000030 | PR | 1 | National Science and Technology Major Project | 2021 |
| Abivertinib | CXHS1800008 | PR | 1 | National Science and Technology Major Project | 2018 |
| Anlotinib | CXHS1700003 | SAP,PR | 2 | New drugs with significant clinical value | 2018 |
| Anlotinib | CXHS1800025 | SAP,PR | 2 | New drugs with significant clinical value | 2018 |
| Anlotinib | CXHS1900040 | PR | 1 | New drugs with significant clinical value | 2021 |
| Orelabrutinib | CXHS1900035 | CA,PR | 2 | New drugs with significant clinical value | 2020 |
| Orelabrutinib | CXHS2000008 | CA,PR | 2 | New drugs with significant clinical value | 2020 |
| Olaparib | JXHS1700046 | PR | 1 | New drugs with significant clinical value | 2019 |
| Olaparib | JXHS1800061 | PR | 1 | New drugs with significant clinical value | 2018 |
| Olaparib | JXHS2100001 | CA,PR | 2 | New drugs with significant clinical value | 2021 |
| Olverembatinib | CXHS2000038 | CA,PR | 2 | New drugs with significant clinical value | 2021 |
| Obinutuzumab | JXSS1900059 | PR | 1 | New drugs with significant clinical value | 2021 |
| Osimertinib | JXHS1700005 | SAP,PR | 2 | New drugs with significant clinical value | 2017 |
| Osimertinib | JXHS1800054 | PR | 1 | New drugs with significant clinical value | 2019 |
| Osimertinib | JXHS2000151 | PR | 1 | New drugs with significant clinical value | 2021 |
| Bevacizumab | CXSS1800017 | PR | 1 | New drugs with significant clinical value | 2018 |
| Bevacizumab | CXSS1900004 | PR | 1 | New drugs with significant clinical value | 2019 |
| Bevacizumab | CXSS2100008 | PR | 1 | New drugs with significant clinical value | 2021 |
| Blinatumomab | JXSS1900060 | PR | 1 | New drugs with significant clinical value | 2020 |
| Pyrotinib | CXHS1700013 | SAP,PR | 2 | New drugs with significant clinical value | 2018 |
| Dalpiciclib | CXHS2101006 | PR,BT | 2 | New drugs with significant clinical value | 2021 |
| Dacomitinib | JXHS1800023 | SAP,PR | 2 | New drugs with significant clinical value | 2019 |
| Dacomitinib | JXHS1900092 | PR | 1 | New drugs with significant clinical value | 2019 |
| Daratumumab | JXSS1800023 | SAP,PR | 2 | New drugs with significant clinical value | 2019 |
| Darolutamide | JXHS2000007 | SAP,PR | 2 | New drugs with significant clinical value | 2021 |
| Dasatinib | JXHL0600105 | SAP | 1 | New drugs with significant clinical value | 2007 |
| Dinutuximab | JXSS2000048 | CA,PR | 2 | New drugs with significant clinical value | 2020 |
| Donafenib | CXHS2000010 | PR | 1 | New drugs with significant clinical value | 2021 |
| Erlotinib | JXHL0500008 | SAP | 1 | New drugs with significant clinical value | 2005 |
| Trastuzumab | JXSS1900012 | PR | 1 | New drugs with significant clinical value | 2020 |
| Ensartinib | CXHS1800045 | CA,PR | 2 | New drugs with significant clinical value | 2020 |
| Envolizumab | CXSS2000060 | CA,PR | 2 | New drugs with significant clinical value | 2021 |
| Enzalutamide | JXHS1900163 | PR | 1 | New drugs with significant clinical value | 2020 |
| Fruquintinib | CXHS1700008 | SAP,PR | 2 | New drugs with significant clinical value | 2018 |
| Furmonertinib | CXHS1900039 | CA,PR | 2 | New drugs with significant clinical value | 2021 |
| Flumatinib | CXHS1800016 | SAP,PR | 2 | New drugs with significant clinical value | 2019 |
| Fluzoparib | CXHS1900033 | SAP,PR | 2 | New drugs with significant clinical value | 2020 |
| Fluzoparib | CXHS2000046 | PR | 1 | Urgently needed new drugs Drugs in short supply | 2021 |
| Gilteritinib | JXHS2000033 | CA,PR | 2 | New drugs with significant clinical value | 2021 |
| Geptanolimab | CXSS2000042 | CA | 1 | New drugs with significant clinical value | 2020 |
| Carfilzomib | JXHS1900150 | CA | 1 | New drugs with significant clinical value | 2021 |
| Camrelizumab | CXSS1800009 | SAP,PR | 2 | New drugs with significant clinical value | 2019 |
| Camrelizumab | CXSS1900023 | PR | 1 | New drugs with significant clinical value | 2020 |
| Camrelizumab | CXSS1900034 | SAP,PR | 2 | New drugs with significant clinical value | 2020 |
| Camrelizumab | CXSS1900035 | SAP,PR | 2 | New drugs with significant clinical value | 2020 |
| Camrelizumab | CXSS2000045 | PR | 1 | New drugs with significant clinical value | 2020 |
| Cladribine | X0403028 | SAP | 1 | New drugs with significant clinical value | 2005 |
| Crizotinib | JXHL1300162 | SAP | 1 | Urgently needed overseas new drugs | 2012 |
| Lenalidomide | CXHS1400266 | PR | 1 | Urgently needed new drugs | 2014 |
| Lenalidomide | JXHS1600024 | SAP,PR | 2 | New drugs with significant clinical value | 2016 |
| Lenalidomide | JXHS1900154 | PR | 1 | New drugs with significant clinical value | 2018 |
| Rituximab | CXSS1900030 | PR | 1 | New drugs with significant clinical value | 2020 |
| Lenvatinib | JXHS1700042 | SAP,PR | 2 | New drugs with significant clinical value | 2018 |
| Mitoxantrone | CXHS2000023 | CA,PR | 2 | New drugs with significant clinical value | 2020 |
| Nivolumab | JXSS1700015 | SAP,PR | 2 | New drugs with significant clinical value | 2018 |
| Nivolumab | JXSS1900001 | PR | 1 | New drugs with significant clinical value | 2017 |
| Nivolumab | JXSS1900037 | PR | 1 | New drugs with significant clinical value | 2020 |
| Nivolumab | JXSS2000053 | CA,PR | 2 | New drugs with significant clinical value | 2021 |
| Niraparib | CXHS2000009 | PR | 1 | New drugs with significant clinical value | 2019 |
| Pembrolizumab | JXSS1800002 | PR | 1 | New drugs with significant clinical value | 2018 |
| Pembrolizumab | JXSS2000010 | PR | 1 | New drugs with significant clinical value | 2020 |
| Pembrolizumab | JXSS2000063 | CA,PR | 2 | New drugs with significant clinical value | 2021 |
| Pamiparib | CXHS2000021 | CA,PR | 2 | New drugs with significant clinical value | 2021 |
| Pertuzumab | JXSS1800020 | PR | 1 | New drugs with significant clinical value | 2019 |
| Palbociclib | JXHL1500268 | CA | 1 | New drugs with significant clinical value | 2018 |
| Palbociclib | JXHL1700152 | PR | 1 | New drugs with significant clinical value | 2018 |
| Pemetrexed | J0401653 | SAP | 1 | Urgently needed overseas new drugs | 2005 |
| Bortezomib | JYHB0500445 | SAP | 1 | New drugs with significant clinical value | 2005 |
| Pralatrexate | JXHS1800080 | CA,PR | 2 | New drugs with significant clinical value | 2020 |
| Pralsetinib | JXHS2000131 | CA,PR | 2 | New drugs with significant clinical value | 2021 |
| Trametinib | JXHS1900091 | PR | 1 | New drugs with significant clinical value | 2020 |
| Trastuzumab | CXSS1900021 | PR | 1 | New drugs with significant clinical value | 2020 |
| Regorafenib | JXHS1500103 | SAP,PR | 2 | New drugs with significant clinical value | 2017 |
| Relmacabtagene Autoleucel | CXSS2000036 | CA,PR | 2 | New drugs with significant clinical value | 2021 |
| Ripretinib | JXHS2000121 | CA,PR | 2 | New drugs with significant clinical value | 2021 |
| Ceritinib | JXHS1700053 | PR | 1 | Innovative drugs with significant clinical advantages | 2018 |
| Savolitinib | CXHS2000012 | CA,PR | 2 | New drugs with significant clinical value | 2020 |
| Sunitinib | JXHL0500229 | SAP | 1 | New drugs with significant clinical value | 2006 |
| Dicycloplatin | CXHS0900153 | SAP | 1 | New drugs with significant clinical value | 2012 |
| Sulfatinib | CXHS1900034 | PR | 1 | New drugs with significant clinical value | 2020 |
| Sorafenib | JXHL0500210 | SAP | 1 | New drugs with significant clinical value | 2005 |
| Sorafenib | JXHS1600040 | PR | 1 | new drugs with significant clinical value | 2017 |
| Toripalimab | CXSS1800006 | PR | 1 | Innovative drugs with significant clinical advantages | 2018 |
| Toripalimab | CXSS2000016 | CA,PR | 2 | New drugs with significant clinical value | 2021 |
| Toripalimab | CXSS2000018 | CA,PR | 2 | New drugs with significant clinical value | 2021 |
| Tislelizumab | CXSS1900025 | SAP,PR | 2 | New drugs with significant clinical value | 2021 |
| Tislelizumab | CXSS2000033 | CA | 1 | New drugs with significant clinical value | 2021 |
| Tislelizumab | CXSS2101004 | CA,PR | 2 | New drugs with significant clinical value | 2021 |
| Tislelizumab | CXSS1800019 | CA,PR | 2 | New drugs with significant clinical value | 2020 |
| Brentuximab | JXSS1900015 | PR | 1 | New drugs with significant clinical value | 2020 |
| Vemurafenib | JXHS1600039 | PR | 1 | New drugs with significant clinical value | 2017 |
| Disitamab | CXSS2000044 | CA,PR | 2 | New drugs with significant clinical value | 2021 |
| Disitamab | CXSS2101011 | CA,PR,BT | 3 | New drugs with significant clinical value | 2021 |
| Chidamide | CXHS1300047 | SAP,CA | 2 | National Science and Technology Major Project | 2014 |
| Chidamide | CXHS1800033 | PR | 1 | Innovative drugs with significantclinical advantages | 2019 |
| Cetuximab | J0402422 | SAP | 1 | New drugs with significant clinical value | 2005 |
| Cetuximab | JXSS1900014 | PR | 1 | New drugs with significant clinical value | 2020 |
| Sintilimab | CXSS1800008 | SAP,PR | 2 | Innovative drugs with significant clinical advantages | 2018 |
| Sintilimab | CXSS2100009 | PR | 1 | New drugs with significant clinical value | 2021 |
| Ibrutinib | JYHB1800207 | PR | 1 | New drugs with significant clinical value | 2019 |
| Imbruvica | JXHS1600066 | PR | 1 | New drugs with significant clinical value | 2017 |
| Inetetamab | CXSS1800023 | PR | 1 | New drugs with significant clinical value | 2020 |
| Ipilimumab | JXSS2000055 | CA,PR | 2 | New drugs with significant clinical value | 2020 |
| Ixazomib | JXHL1600072 | SAP,PR | 2 | New drugs with significant clinical value | 2018 |
| Inotuzumab ozogamicin | JXSS2000001 | PR | 1 | New drugs with significant clinical value | 2021 |
| Utidelone | CXHS1800005 | SAP,PR | 2 | New drugs with significant clinical value | 2021 |
| Zanubrutinib | CXHS1800024 | SAP,PR | 2 | New drugs with significant clinical value | 2020 |
| Zanubrutinib | CXHS1800030 | SAP,PR | 2 | New drugs with significant clinical value | 2020 |
| Zanubrutinib | CXHS2000037 | CA,PR | 2 | New drugs with significant clinical value | 2021 |
| Decitabine | JXHL0700089 | SAP | 1 | Urgently needed overseas new drugs | 2007 |
| Anastrozole | CYHS1800473 | PR | 1 | Generic | 2019 |
| Anastrozole | CYHS1900269 | PR | 1 | Generic | 2019 |
| Azacitidine | CYHS1600206 | PR | 1 | Generic | 2017 |
| Paclitaxel(Albumin Bound) | CYHS1790004 | PR | 1 | Generic | 2018 |
| Bendamustine | CYHS1900557 | PR | 1 | Generic | 2020 |
| Docetaxel | CYHS1700558 | PR | 1 | Generic | 2019 |
| Erlotinib | CYHS1790011 | SAP | 1 | Generic | 2019 |
| Fulvestrant | CYHS1900083 | PR | 1 | Generic | 2020 |
| Gefitinib | CYHS1490010 | PR | 1 | Generic | 2016 |
| Letrozole | CYHS1900655 | PR | 1 | Generic | 2019 |
| Pemetrexed | CYHS1600043 | PR | 1 | Generic | 2018 |
| Pemetrexed | CYHS1800165 | PR | 1 | Generic | 2020 |
| Pemetrexed | CYHS1900295 | PR | 1 | Generic | 2019 |
| Bortezomib | CYHS1900843 | PR | 1 | Generic | 2017 |
| Sorafenib | CYHS1900700 | PR | 1 | Generic | 2019 |
| Temozolomide | CYHS1700055 | PR | 1 | Generic | 2018 |
| Imatinib | CYHS1700601 | PR | 1 | Generic | 2018 |
| Paclitaxel(Albumin Bound) | CYHS1600152 | PR | 1 | Generic | 2018 |
| Paclitaxel | CYHS1900682 | PR | 1 | Generic | 2020 |

Note: **SAP:** Special Approval. **PR:** Priority Review. **CA:** Conditional Approval. **BT:** Breakthrough Therapy

## eTable S2: Supplement to Table 1

| Indication characteristics | Types | Indications  No (%) | Time for review  (days) | | P value |
| --- | --- | --- | --- | --- | --- |
| Solid cancer in the initial indications | Thoracic malignancies | 35(25.0%) | 289 | 0.281 | |
|  | Breast cancer | 19(13.2%) | 418 |  |  |
|  | Gastrointestinal cancers | 19(13.2%) | 295 |  |  |
|  | Genitourinary cancers | 13(9.0%) | 287 |  |  |
|  | Head and neck cancer | 8(5.6%) | 289 |  |  |
|  | Gynecological malignancies | 4(2.8%) | 280 |  |  |
|  | Skin cancers | 4(2.8%) | 257.5 |  |  |
|  | Non-cancerous soft-tissue tumor | 1(0.7%) | 299 |  |  |
|  | Brain tumors | 1(0.7%) | 552 |  |  |
|  | Others | 2(1.4%) | 396 |  |  |
| Other Mechanism of action | Poly-ADP-ribose polymerase inhibitor(ADP) | 7(4.9%) | 265 | 0.236 | |
|  | Cyclin dependent kinase4/6(CDK4/6) | 4(2.8%) | 384 |  |  |
|  | Proteasome inhibitor(PI) | 2(1.4%) | 454.5 |  |  |
|  | Histone deacetylase inhibitor (HDACI) | 2(1.4%) | 519 |  |  |
|  | Chimeric Antigen Receptor T-Cell Immunotherapy (CAR-T) | 2(1.4%) | 457 |  |  |
|  | Dihydrofolate reductase (DHFR) | 1(0.7%) | 607 |  |  |
| Monoclonal antibodies target class^d^ | PD1/PD-L1 | 25（45.5%） | 269.5 | **0.012** | |
|  | VEGF/VEGFR | 12（21.8%） | 424.5 |  |  |
|  | HER2 | 6（10.9%） | 313 |  |  |
|  | CD19 | 3（5.5%） | 430 |  |  |
|  | CD20 | 2（3.6%） | 542.5 |  |  |
|  | EGFR | 2（3.6%） | 347.5 |  |  |
|  | CD38 | 1（1.8%） | 258 |  |  |
|  | GD2 | 1（1.8%） | 286 |  |  |
|  | CD30 | 1（1.8%） | 382 |  |  |
|  | CTLA-4 | 1（1.8%） | 190 |  |  |
|  | CD22 | 1（1.8%） | 700 |  |  |
| Medical insurance catalog | Class B^a^ | 38(26.4%) | 473 | **0.035** | |
|  | Class B and NRND^b^ | 76(52.8%) | 317 |  |  |
|  | Class A^c^ | 1(0.7%) | 439 |  |  |

Note: ^a^ Class B: Class B National Medical Insurance Drug Directory; ^b^ NRND: China’s National Reimbursement Negotiation Drug List; ^c^ Class A: Class A National Medical Insurance Drug Directory; ^d^Monoclonal antibodies target class: This section contains only drugs that possess monoclonal antibodies.

## eTable S3: Review and approval of EP oncology drugs from 2005-2021

| **Drug Name** | **Handling No.** | **Marketing Authorization Holder** | **Review type** | **Approval Type** | **Initial indications** | **Mechanism of Action** | **NME or novel biologic** | **Review conclusion** | **Time consumed (days)** | **Medical insurance catalog** |
| --- | --- | --- | --- | --- | --- | --- | --- | --- | --- | --- |
| Abemaciclib | JXHS1900144 | Foreign | NDA | Novel | Breast Cancer | CDK4/6 inhibitor | Chemical | Approved | 418 | Class B, NRND |
| Abiraterone | JXHS1800011 | Foreign | NDA | Novel | Genitourinary Cancers | Endocrine therapy | Chemical | Approved | 257 | Class B |
| Avapritinib | JXHS2000050 | Domestic | NDA | Novel | Gastrointestinal Cancers | TKI | Chemical | Approved | 336 | None |
| Afatinib | JXHS1600009 | Foreign | NDA | Novel | Thoracic Malignancies | TKI | Chemical | Approved | 365 | Class B |
| Axicabtagene ciloleucel | CXSS2000006 | Domestic | NDA | Novel | Haematological Malignancies | CAR-T | Biologic | Approved | 484 | None |
| Alectinib | JXHS1800001 | Foreign | NDA | Novel | Thoracic Malignancies | TKI | Chemical | Approved | 194 | Class B,NRND |
| Almonertinib | CXHS1900011 | Domestic | NDA | Novel | Thoracic Malignancies | TKI | Chemical | Approved | 215 | Class B,NRND |
| Almonertinib | CXHS2101017 | Domestic | NDA | Supplemental | Thoracic Malignancies | TKI | Chemical | Approved | 336 | Class B,NRND |
| Apalutamide | JXHS1900036 | Foreign | NDA | Novel | Genitourinary Cancers | Endocrine therapy | Chemical | Approved | 179 | None |
| Apalutamide | JXHS1900156 | Foreign | NDA | Supplemental | Genitourinary Cancers | Endocrine therapy | Chemical | Approved | 258 | Class B,NRND |
| Apatinib | CXHS1100133 | Domestic | NDA | Novel | Gastrointestinal Cancers | TKI | Chemical | Approved | 1179 | Class B,NRND |
| Atezolizumab | JXSS2000002 | Foreign | NDA | Supplemental | Gastrointestinal Cancers | Monoclonal antibody | Biologic | Approved | 257 | None |
| Atezolizumab | JXSS2000033 | Foreign | NDA | Supplemental | Thoracic Malignancies | Monoclonal antibody | Biologic | Approved | 218 | None |
| Axitinib | JXHS1400080 | Foreign | NDA | Novel | Genitourinary Cancers | TKI | Chemical | Approved | 270 | Class B |
| Icotinib | CXHL0502177 | Domestic | NDA | Novel | Thoracic Malignancies | TKI | Chemical | Approved | 294 | Class B,NRND |
| Icotinib | CXHS2000030 | Domestic | NDA | Supplemental | Thoracic Malignancies | TKI | Chemical | Approved | 258 | Class B,NRND |
| Abivertinib | CXHS1800008 | Domestic | NDA | Novel | Thoracic Malignancies | TKI | Chemical | Not approved | NA | NA |
| Anlotinib | CXHS1700003 | Domestic | NDA | Novel | Thoracic Malignancies | TKI | Chemical | Approved | 427 | Class B,NRND |
| Anlotinib | CXHS1800025 | Domestic | NDA | Supplemental | Non-cancerous soft-tissue tumour | TKI | Chemical | Approved | 299 | Class B,NRND |
| Anlotinib | CXHS1900040 | Domestic | NDA | Supplemental | Head and neck cancer | TKI | Chemical | Approved | 418 | Class B,NRND |
| Orelabrutinib | CXHS1900035 | Domestic | NDA | Novel | Haematological Malignancies | TKI | Chemical | Approved | 399 | Class B,NRND |
| Orelabrutinib | CXHS2000008 | Domestic | NDA | Supplemental | Haematological Malignancies | TKI | Chemical | Approved | 289 | Class B,NRND |
| Olaparib | JXHS1700046 | Foreign | NDA | Novel | Gynaecological Malignancies | ADP | Chemical | Approved | 265 | Class B,NRND |
| Olaparib | JXHS1800061 | Foreign | NDA | Novel | Gynaecological Malignancies | ADP | Chemical | Approved | 387 | Class B,NRND |
| Olaparib | JXHS2100001 | Foreign | NDA | Supplemental | Genitourinary Cancers | ADP | Chemical | Approved | 160 | Class B,NRND |
| Olverembatinib | CXHS2000038 | Domestic | NDA | Novel | Haematological Malignancies | TKI | Chemical | Approved | 415 | Class B,NRND |
| Obinutuzumab | JXSS1900059 | Foreign | NDA | Novel | Haematological Malignancies | Monoclonal antibody | Biologic | Approved | 615 | Class B,NRND |
| Osimertinib | JXHS1700005 | Foreign | NDA | Novel | Thoracic Malignancies | TKI | Chemical | Approved | 49 | Class B,NRND |
| Osimertinib | JXHS1800054 | Foreign | NDA | Novel | Thoracic Malignancies | TKI | Chemical | Approved | 309 | Class B,NRND |
| Osimertinib | JXHS2000151 | Foreign | NDA | Supplemental | Thoracic Malignancies | TKI | Chemical | Approved | 195 | Class B,NRND |
| Bevacizumab | CXSS1800017 | Domestic | NDA | Novel | Thoracic Malignancies | Monoclonal antibody | Biologic | Approved | 483 | Class B |
| Bevacizumab | CXSS1900004 | Domestic | NDA | Novel | Thoracic Malignancies | Monoclonal antibody | Biologic | Approved | 495 | Class B |
| Bevacizumab | CXSS2100008 | Domestic | NDA | Supplemental | Gastrointestinal Cancers | Monoclonal antibody | Biologic | Approved | 163 | Class B |
| Blinatumomab | JXSS1900060 | Foreign | NDA | Novel | Haematological Malignancies | Monoclonal antibody | Biologic | Approved | 406 | None |
| Pyrotinib | CXHS1700013 | Domestic | NDA | Novel | Breast Cancer | TKI | Chemical | Approved | 358 | Class B,NRND |
| Dalpiciclib | CXHS2101006 | Domestic | NDA | Novel | Breast Cancer | CDK4/6 inhibitor | Chemical | Approved | 255 | Class B,NRND |
| Dacomitinib | JXHS1800023 | Foreign | NDA | Novel | Thoracic Malignancies | TKI | Chemical | Approved | 361 | Class B,NRND |
| Dacomitinib | JXHS1900092 | Foreign | NDA | Novel | Skin Cancers | TKI | Chemical | Approved | 239 | Class B,NRND |
| Daratumumab | JXSS1800023 | Foreign | NDA | Novel | Haematological Malignancies | Monoclonal antibody | Biologic | Approved | 258 | None |
| Darolutamide | JXHS2000007 | Foreign | NDA | Novel | Genitourinary Cancers | Endocrine therapy | Chemical | Approved | 354 | Class B,NRND |
| Dasatinib | JXHL0600105 | Foreign | NDA | Novel | Haematological Malignancies | TKI | Chemical | Approved | 289 | Class B |
| Dinutuximab | JXSS2000048 | Domestic | NDA | Novel | Head and neck cancer | Monoclonal antibody | Biologic | Approved | 286 | None |
| Donafenib | CXHS2000010 | Domestic | NDA | Novel | Gastrointestinal Cancers | TKI | Chemical | Approved | 389 | Class B,NRND |
| Erlotinib | JXHL0500008 | Foreign | NDA | Novel | Thoracic Malignancies | TKI | Chemical | Approved | 226 | Class B |
| Trastuzumab | JXSS1900012 | Foreign | NDA | Novel | Breast Cancer | Monoclonal antibody | Biologic | Approved | 301 | Class B,NRND |
| Ensartinib | CXHS1800045 | Domestic | NDA | Novel | Thoracic Malignancies | TKI | Chemical | Approved | 692 | Class B,NRND |
| Envolizumab | CXSS2000060 | Domestic | NDA | Novel | Gastrointestinal Cancers | Monoclonal antibody | Biologic | Approved | 343 | None |
| Enzalutamide | JXHS1900163 | Foreign | NDA | Novel | Genitourinary Cancers | Endocrine therapy | Chemical | Approved | 334 | Class B,NRND |
| Fruquintinib | CXHS1700008 | Domestic | NDA | Novel | Gastrointestinal Cancers | TKI | Biologic | Approved | 440 | Class B,NRND |
| Furmonertinib | CXHS1900039 | Domestic | NDA | Novel | Thoracic Malignancies | TKI | Chemical | Approved | 454 | Class B,NRND |
| Flumatinib | CXHS1800016 | Domestic | NDA | Novel | Haematological Malignancies | TKI | Chemical | Approved | 500 | Class B,NRND |
| Fluzoparib | CXHS1900033 | Domestic | NDA | Novel | Breast Cancer | ADP | Biologic | Approved | 419 | Class B,NRND |
| Fluzoparib | CXHS2000046 | Domestic | NDA | Supplemental | Breast Cancer | ADP | Biologic | Approved | 182 | Class B,NRND |
| Gilteritinib | JXHS2000033 | Foreign | NDA | Novel | Haematological Malignancies | TKI | Chemical | Approved | 298 | None |
| Geptanolimab | CXSS2000042 | Domestic | NDA | Novel | Haematological Malignancies | Monoclonal antibody | Chemical | Not approved | NA | NA |
| Carfilzomib | JXHS1900150 | Foreign | NDA | Novel | Haematological Malignancies | PI | Chemical | Approved | 279 | Class B,NRND |
| Camrelizumab | CXSS1800009 | Domestic | NDA | Novel | Haematological Malignancies | Monoclonal antibody | Biologic | Approved | 401 | Class B,NRND |
| Camrelizumab | CXSS1900023 | Domestic | NDA | Supplemental | Gastrointestinal Cancers | Monoclonal antibody | Biologic | Approved | 295 | Class B,NRND |
| Camrelizumab | CXSS1900034 | Domestic | NDA | Supplemental | Thoracic Malignancies | Monoclonal antibody | Biologic | Approved | 284 | Class B,NRND |
| Camrelizumab | CXSS1900035 | Domestic | NDA | Supplemental | Thoracic Malignancies | Monoclonal antibody | Biologic | Approved | 284 | Class B,NRND |
| Camrelizumab | CXSS2000045 | Domestic | NDA | Supplemental | Head and neck cancer | Monoclonal antibody | Biologic | Approved | 211 | Class B,NRND |
| Cladribine | X0403028 | Domestic | NDA | Novel | Haematological Malignancies | Cytotoxic drug | Chemical | Approved | 546 | None |
| Crizotinib | JXHL1300162 | Foreign | IND | Novel | Thoracic Malignancies | TKI | Chemical | Approved | 233 | Class B,NRND |
| Lenalidomide | CXHS1400266 | Domestic | NDA | Novel | Haematological Malignancies | Cytotoxic drug | Chemical | Approved | 1046 | Class B |
| Lenalidomide | JXHS1600024 | Domestic | NDA | Novel | Haematological Malignancies | Cytotoxic drug | Chemical | Approved | 700 | Class B |
| Lenalidomide | JXHS1900154 | Domestic | NDA | Supplemental | Haematological Malignancies | Cytotoxic drug | Chemical | Approved | 476 | Class B |
| Rituximab | CXSS1900030 | Domestic | NDA | Novel | Haematological Malignancies | Monoclonal antibody | Biologic | Approved | 470 | Class B |
| Lenvatinib | JXHS1700042 | Foreign | NDA | Novel | Gastrointestinal Cancers | TKI | Chemical | Approved | 311 | Class B |
| Mitoxantrone | CXHS2000023 | Domestic | NDA | Novel | Haematological Malignancies | Cytotoxic drug | Chemical | Approved | 138 | Class B,NRND |
| Nivolumab | JXSS1700015 | Foreign | NDA | Novel | Thoracic Malignancies | Monoclonal antibody | Biologic | Approved | 226 | None |
| Nivolumab | JXSS1900001 | Foreign | NDA | Novel | Head and neck cancer | Monoclonal antibody | Biologic | Approved | 246 | None |
| Nivolumab | JXSS1900037 | Foreign | NDA | Supplemental | Gastrointestinal Cancers | Monoclonal antibody | Biologic | Approved | 263 | None |
| Nivolumab | JXSS2000053 | Foreign | NDA | Supplemental | Thoracic Malignancies | Monoclonal antibody | Biologic | Approved | 180 | None |
| Niraparib | CXHS2000009 | Domestic | NDA | Novel | Gynaecological Malignancies | ADP | Chemical | Approved | 180 | Class B,NRND |
| Pembrolizumab | JXSS1800002 | Foreign | NDA | Novel | Skin Cancers | Monoclonal antibody | Biologic | Approved | 165 | None |
| Pembrolizumab | JXSS2000010 | Foreign | NDA | Supplemental | Head and neck cancer | Monoclonal antibody | Biologic | Approved | 224 | None |
| Pembrolizumab | JXSS2000063 | Foreign | NDA | Supplemental | Gastrointestinal Cancers | Monoclonal antibody | Biologic | Approved | 224 | None |
| Pamiparib | CXHS2000021 | Domestic | NDA | Novel | Gynaecological Malignancies | ADP | Chemical | Approved | 295 | Class B,NRND |
| Pertuzumab | JXSS1800020 | Foreign | NDA | Novel | Breast Cancer | TKI | Biologic | Approved | 325 | Class B,NRND |
| Palbociclib | JXHL1500268 | Foreign | CTA | Novel | Breast Cancer | CDK4/6 inhibitor | Chemical | Approved | 1079 | Class B,NRND |
| Palbociclib | JXHL1700152 | Foreign | CTA | Novel | Breast Cancer | CDK4/6 inhibitor | Chemical | Approved | 350 | Class B |
| Pemetrexed | J0401653 | Foreign | NDA | Novel | Thoracic Malignancies | Cytotoxic drug | Chemical | Approved | 423 | Class B |
| Bortezomib | JYHB0500445 | Foreign | NDA | Novel | Haematological Malignancies | Cytotoxic drug | Chemical | Approved | 275 | Class B |
| Pralatrexate | JXHS1800080 | Foreign | NDA | Novel | Haematological Malignancies | DHFR | Chemical | Approved | 607 | None |
| Pralsetinib | JXHS2000131 | Domestic | NDA | Novel | Thoracic Malignancies | TKI | Chemical | Approved | 198 | None |
| Trametinib | JXHS1900091 | Foreign | NDA | Novel | Thoracic Malignancies | TKI | Chemical | Approved | 239 | Class B,NRND |
| Trastuzumab | CXSS1900021 | Domestic | NDA | Novel | Breast Cancer | Monoclonal antibody | Biologic | Approved | 478 | Class B,NRND |
| Regorafenib | JXHS1500103 | Foreign | NDA | Novel | Gastrointestinal Cancers | TKI | Chemical | Approved | 472 | Class B,NRND |
| Relmacabtagene Autoleucel | CXSS2000036 | Domestic | NDA | Novel | Haematological Malignancies | CAR-T | Biologic | Approved | 430 | None |
| Ripretinib | JXHS2000121 | Domestic | NDA | Novel | Gastrointestinal Cancers | TKI | Chemical | Approved | 251 | Class B,NRND |
| Ceritinib | JXHS1700053 | Foreign | NDA | Novel | Thoracic Malignancies | TKI | Chemical | Approved | 178 | Class B,NRND |
| Savolitinib | CXHS2000012 | Domestic | NDA | Novel | Thoracic Malignancies | TKI | Chemical | Approved | 383 | Class B,NRND |
| Sunitinib | JXHL0500229 | Foreign | NDA | Novel | Genitourinary Cancers | TKI | Chemical | Approved | 187 | Class B |
| Dicycloplatin | CXHS0900153 | Domestic | NDA | Novel | Genitourinary Cancers | Cytotoxic drug | Chemical | Not Approved | 584 | NA |
| Sulfatinib | CXHS1900034 | Domestic | NDA | Novel | Gastrointestinal Cancers | TKI | Chemical | Approved | 422 | Class B,NRND |
| Sorafenib | JXHL0500210 | Foreign | NDA | Novel | Genitourinary Cancers | TKI | Chemical | Approved | 333 | Class B |
| Sorafenib | JXHS1600040 | Foreign | NDA | Novel | Head and neck cancer | TKI | Chemical | Approved | 349 | Class B |
| Toripalimab | CXSS1800006 | Domestic | NDA | Novel | Skin Cancers | Monoclonal antibody | Biologic | Approved | 276 | Class B,NRND |
| Toripalimab | CXSS2000016 | Domestic | NDA | Supplemental | Head and neck cancer | Monoclonal antibody | Biologic | Approved | 292 | Class B,NRND |
| Toripalimab | CXSS2000018 | Domestic | NDA | Supplemental | Genitourinary Cancers | Monoclonal antibody | Biologic | Approved | 329 | Class B,NRND |
| Tislelizumab | CXSS1900025 | Domestic | NDA | Supplemental | Genitourinary Cancers | Monoclonal antibody | Biologic | Approved | 316 | Class B,NRND |
| Tislelizumab | CXSS2000033 | Domestic | NDA | Supplemental | Gastrointestinal Cancers | Monoclonal antibody | Biologic | Approved | 285 | Class B,NRND |
| Tislelizumab | CXSS2101004 | Domestic | NDA | Supplemental | Solid cancer | Monoclonal antibody | Biologic | Approved | 285 | Class B,NRND |
| Tislelizumab | CXSS1800019 | Domestic | NDA | Novel | Haematological Malignancies | Monoclonal antibody | Biologic | Approved | 487 | Class B,NRND |
| Brentuximab | JXSS1900015 | Foreign | NDA | Novel | Haematological Malignancies | Monoclonal antibody | Biologic | Approved | 382 | Class B,NRND |
| Vemurafenib | JXHS1600039 | Foreign | NDA | Novel | Skin Cancers | TKI | Chemical | Approved | 345 | Class B,NRND |
| Disitamab | CXSS2000044 | Domestic | NDA | Novel | Gastrointestinal Cancers | Monoclonal antibody | Chemical | Approved | 287 | None |
| Disitamab | CXSS2101011 | Domestic | NDA | Supplemental | Genitourinary Cancers | Monoclonal antibody | Chemical | Approved | 287 | None |
| Chidamide | CXHS1300047 | Domestic | NDA | Novel | Haematological Malignancies | HDACI | Chemical | Approved | 657 | Class B,NRND |
| Chidamide | CXHS1800033 | Domestic | NDA | Novel | Breast Cancer | HDACI | Chemical | Approved | 381 | Class B,NRND |
| Cetuximab | J0402422 | Foreign | NDA | Novel | Gastrointestinal Cancers | Monoclonal antibody | Biologic | Approved | 377 | Class B,NRND |
| Cetuximab | JXSS1900014 | Foreign | NDA | Novel | Head and neck cancer | TKI | Biologic | Approved | 318 | Class B,NRND |
| Sintilimab | CXSS1800008 | Domestic | NDA | Novel | Haematological Malignancies | Monoclonal antibody | Biologic | Approved | 255 | Class B,NRND |
| Sintilimab | CXSS2100009 | Domestic | NDA | Supplemental | Gastrointestinal Cancers | Monoclonal antibody | Biologic | Approved | 163 | Class B,NRND |
| Ibrutinib | JYHB1800207 | Domestic | NDA | Supplemental | Haematological Malignancies | TKI | Chemical | Approved | 249 | Class B,NRND |
| Imbruvica | JXHS1600066 | Foreign | NDA | Novel | Haematological Malignancies | TKI | Chemical | Approved | 294 | Class B,NRND |
| Inetetamab | CXSS1800023 | Domestic | NDA | Supplemental | Breast Cancer | Monoclonal antibody | Biologic | Approved | 651 | Class B,NRND |
| Ipilimumab | JXSS2000055 | Foreign | NDA | Novel | Thoracic Malignancies | Monoclonal antibody | Biologic | Approved | 190 | None |
| Ixazomib | JXHL1600072 | Foreign | CTA | Novel | Haematological Malignancies | PI | Chemical | Approved | 630 | Class B |
| Inotuzumab ozogamicin | JXSS2000001 | Foreign | NDA | Novel | Haematological Malignancies | Monoclonal antibody | Biologic | Approved | 700 | None |
| Utidelone | CXHS1800005 | Domestic | NDA | Novel | Breast Cancer | Cytotoxic drug | Chemical | Approved | 1074 | Class B,NRND |
| Zanubrutinib | CXHS1800024 | Domestic | NDA | Novel | Haematological Malignancies | TKI | Chemical | Approved | 651 | Class B,NRND |
| Zanubrutinib | CXHS1800030 | Domestic | NDA | Supplemental | Haematological Malignancies | TKI | Chemical | Approved | 595 | Class B,NRND |
| Zanubrutinib | CXHS2000037 | Domestic | NDA | Supplemental | Haematological Malignancies | TKI | Chemical | Approved | 267 | Class B,NRND |
| Decitabine | JXHL0700089 | Foreign | NDA | Novel | Haematological Malignancies | Cytotoxic drug | Chemical | Approved | 540 | Class B |
| Anastrozole | CYHS1800473 | Domestic | ANDA | / | Breast Cancer | Endocrine therapy | Chemical | Approved | 587 | Class B |
| Anastrozole | CYHS1900269 | Domestic | ANDA | / | Breast Cancer | Endocrine therapy | Chemical | Approved | 1139 | Class B |
| Azacitidine | CYHS1600206 | Domestic | ANDA | / | Haematological Malignancies | Cytotoxic drug | Chemical | Approved | 868 | Class B |
| Paclitaxel(Albumin Bound) | CYHS1790004 | Domestic | ANDA | / | Breast Cancer | Cytotoxic drug | Chemical | Approved | 120 | Class B |
| Bendamustine | CYHS1900557 | Domestic | ANDA | / | Haematological Malignancies | Cytotoxic drug | Chemical | Approved | 532 | Class B |
| Docetaxel | CYHS1700558 | Domestic | ANDA | / | Solid cancer | Cytotoxic drug | Chemical | Approved | 507 | Class B |
| Erlotinib | CYHS1790011 | Domestic | ANDA | / | Thoracic Malignancies | TKI | Chemical | Approved | 941 | Class B |
| Fulvestrant | CYHS1900083 | Domestic | ANDA | / | Breast Cancer | Endocrine therapy | Chemical | Approved | 571 | Class B |
| Gefitinib | CYHS1490010 | Domestic | ANDA | / | Thoracic Malignancies | TKI | Chemical | Approved | 1067 | Class B |
| Letrozole | CYHS1900655 | Domestic | ANDA | / | Breast Cancer | Endocrine therapy | Chemical | Approved | 581 | Class B |
| Pemetrexed | CYHS1600043 | Domestic | ANDA | / | Thoracic Malignancies | Cytotoxic drug | Chemical | Approved | 435 | Class B |
| Pemetrexed | CYHS1800165 | Domestic | ANDA | / | Thoracic Malignancies | Cytotoxic drug | Chemical | Approved | 262 | Class B |
| Pemetrexed | CYHS1900295 | Domestic | ANDA | / | Thoracic Malignancies | Cytotoxic drug | Chemical | Approved | 597 | Class B |
| Bortezomib | CYHS1900843 | Domestic | ANDA | / | Haematological Malignancies | Cytotoxic drug | Chemical | Not approved | 679 | NA |
| Sorafenib | CYHS1900700 | Domestic | ANDA | / | Gastrointestinal Cancers | TKI | Chemical | Approved | 65 | Class B |
| Temozolomide | CYHS1700055 | Domestic | ANDA | / | Brain Tumours | TKI | Chemical | Approved | 552 | Class B |
| Imatinib | CYHS1700601 | Domestic | ANDA | / | Haematological Malignancies | TKI | Chemical | Approved | 1191 | Class B |
| Paclitaxel(Albumin Bound) | CYHS1600152 | Domestic | ANDA | / | Breast Cancer | Cytotoxic drug | Chemical | Approved | 187 | Class B |
| Paclitaxel | CYHS1900682 | Domestic | ANDA | / | Thoracic Malignancies | Cytotoxic drug | Chemical | Approved | 439 | Class A |

Note: **ANDA:** Abbreviated New Drug Application. **NDA:** New Drug Application. **IND:** Investigational New Drug. **CTA:** Clinical Trial Authorization.

**TKI:** Tyrosine kinase inhibitor. **ADP:** Poly-ADP-ribose polymerase inhibitor. **CDK4/6:** Cyclin dependent kinase4/6. **PI：**Proteasome inhibitor. **HDACI :**Histone deacetylase inhibitor. **CAR-T:** Chimeric Antigen Receptor T-Cell Immunotherapy. **DHFR:** Dihydrofolate reductase.

**ClassB:** Class B National Medical Insurance Drug Directory. **NRND:** China’s National Reimbursement Drug List. **ClassA:** Class A National Medical Insurance Drug Directory.

## eTable S4: EP approval drugs with approval times of less than 200 days and more than 1,000 days

| **Handling No.** | **Drug Name** | **Time consumed (days)** |
| --- | --- | --- |
| JXHS1700005 | Osimertinib | 49 |
| CYHS1900700 | Sorafenib | 65 |
| CYHS1790004 | Paclitaxel(Albumin Bound) | 120 |
| CXHS2000023 | Mitoxantrone | 138 |
| JXHS2100001 | Olaparib | 160 |
| CXSS2100008 | Bevacizumab | 163 |
| CXSS2100009 | Sintilimab | 163 |
| JXSS1800002 | Pembrolizumab | 165 |
| JXHS1700053 | Ceritinib | 178 |
| JXHS1900036 | Apalutamide | 179 |
| JXSS2000053 | Nivolumab | 180 |
| CXHS2000009 | Niraparib | 180 |
| CXHS2000046 | Fluzoparib | 182 |
| JXHL0500229 | Sunitinib | 187 |
| CYHS1600152 | Paclitaxel (Albumin Bound) | 187 |
| JXSS2000055 | Ipilimumab | 190 |
| JXHS1800001 | Alectinib | 194 |
| JXHS2000151 | Osimertinib | 195 |
| JXHS2000131 | Pralsetinib | 198 |
| CXHS1100133 | Apatinib | 1179 |
| CXHS1400266 | Lenalidomide | 1046 |
| JXHL1500268 | Palbociclib | 1079 |
| CXHS1800005 | Utidelone | 1074 |
| CYHS1900269 | Anastrozole | 1139 |
| CYHS1490010 | Gefitinib | 1067 |
| CYHS1700601 | Iimatinib | 1191 |

## eTable S5: Characteristics of the pivotal trials of oncology indications approved for EP approval during 2005-2021

| **Genetic name** | **Handling No.** | **NCT ID or CTR ID** | **Initial indications** | **Randomization** | **Design type** | **Degree of blindness** | **Trials Phase** | **Primary outcome** | **Primary outcome value** | **HR** | **Trial center** | **MCBS** |
| --- | --- | --- | --- | --- | --- | --- | --- | --- | --- | --- | --- | --- |
| Abemaciclib | JXHS1900144 | NCT03155997 | Breast Cancer | Randomized | Parallel | Double-blinded | Ⅲ | PFS | 28.18 ms vs. 14.76 ms | 0.54(0.418-0.698) | IMCT | N(3) |
| Abemaciclib | JXHS1900144 | NCT02763566^b^ | Breast Cancer | Randomized | Parallel | Open | Ⅲ | PFS | Not yet reached vs. 13.28 ms |  | IMCT |  |
| Abiraterone | JXHS1800011 | NCT01715285^c^ | Genitourinary Cancers | Randomized | Parallel | Open | Ⅲ | OS | Not yet reached vs34.7ms | 0.621(0.509-0.756) | IMCT | Y(4) |
| Avapritinib | JXHS2000050 | NCT02508532 | Gastrointestinal Cancers | Non-randomized | Singe-arm | Open | Ⅱ | ORR | 89% | DOR Not yet reached | MCCT | N(3) |
| Avapritinib | JXHS2000050 | NCT04254939^b^ | Gastrointestinal Cancers | Non-randomized | Singe-arm | Open | Ⅱ | ORR | 62.50% |  | MCCT |  |
| Afatinib | JXHS1600009 | NCT00949650 |  | Randomized | Parallel | Open | Ⅲ | OS | 33.3ms vs. 21.2ms |  | IMCT | Y(5) |
| Afatinib | JXHS1600009 | NCT01121393^b^ | Thoracic Malignancies | Randomized | Parallel | Open | Ⅲ | PFS | 13.73ms vs. 5.55ms | 0.261(0.192-0.355) | IMCT |  |
| Axicabtagene ciloleucel | CXSS2000006 | NCT02348216 | Haematological Malignancies | Non-randomized | Singe-arm | Open | Ⅰ and Ⅱ | ORR | 74% | DOR Not yet reached | IMCT |  |
| Axicabtagene ciloleucel | CXSS2000006 | NA/CTR20181687^b^ | Haematological Malignancies | Non-randomized | Singe-arm | Open | Ⅰ and Ⅱ | ORR | 79.2% |  | MCCT |  |
| Alectinib | JXHS1800001 | NCT02075840^c^ | Thoracic Malignancies | Randomized | Parallel | Open | Ⅲ | PFS | 34.8ms vs. 10.9ms | 0.43(0.32-0.58) | IMCT | Y(4) |
| Almonertinib | CXHS1900011 | NCT02981108 | Thoracic Malignancies | Non-randomized | Singe-arm | Open | Ⅱ | ORR | 68.90% |  | MCCT | S(3) |
| Almonertinib | CXHS2101017 | NCT03849768 | Thoracic Malignancies | Randomized | Parallel | Double-blinded | Ⅱ | PFS | 19.3ms vs.9.9ms | 0.463(0.359-0.596) | MCCT | N(3) |
| Apalutamide | JXHS1900036 | NCT01946204^c^ | Genitourinary Cancers | Randomized | Parallel | Double-blinded | Ⅲ | OS | 52.2ms vs24.1ms | 0.65(0.53-0.79) | IMCT | N(3) |
| Apalutamide | JXHS1900156 | NCT02489318^c^ | Genitourinary Cancers | Randomized | Parallel | Double-blinded | Ⅲ | MFS | Not yet reached vs.22.08ms | 0.48(0.39-0.60) | IMCT | N(4) |
| Apatinib | CXHS1100133 | NCT00970138 | Gastrointestinal Cancers | Randomized | Parallel | Double-blinded | Ⅱ | OS | 4.83ms |  | MCCT | N(2) |
| Atezolizumab | JXSS2000002 | NCT03434379^c^ | Gastrointestinal Cancers | Randomized | Parallel | Open | Ⅲ | OS | 15 ms vs.10.2ms | 0.8 (0.68- 0.95) | IMCT | N(3) |
| Atezolizumab | JXSS2000033 | NCT02409342 | Thoracic Malignancies | Randomized | Parallel | Open | Ⅲ | OS | 20.2ms vs.13.1ms | 0.59(0.40,0.89) | IMCT | N(3) |
| Axitinib | JXHS1400080 | NCT00678392^c^ | Genitourinary Cancers | Randomized | Parallel | Open | Ⅲ | PFS | 6.7ms vs.4.7ms | 0.572(0.359-0.913) | IMCT | Y(4) |
| Icotinib | CXHL0502177 | NA/ChiCTR-TRC-09000506 | Thoracic Malignancies | Randomized | Parallel | Double-blinded | Ⅲ | PFS | 5ms |  | MCCT | Y(4) |
| Icotinib | CXHS2000030 | NCT02448797 | Thoracic Malignancies | Randomized | Parallel | Open | Ⅲ | DFS | 46.95ms vs. 22.11ms | 0.36(0.24- | MCCT | Y(4) |
|  |  |  |  |  |  |  |  |  |  | 0.55) |  |  |
| Anlotinib | CXHS1700003 | NCT03452592 | Thoracic Malignancies | Randomized | Parallel | Double-blinded | Ⅱ | OS | 9.63ms vs.6.30ms | 0.70(0.55-0.89) | MCCT | Y(5) |
| Anlotinib | CXHS1800025 | NCT02449343 | Non-cancerous soft-tissue tumour | Randomized | Parallel | Double-blinded | Ⅲ | PFS | 6.2ms vs.1.5ms | 0.33(0.23-0.47) | MCCT | S(3) |
| Anlotinib | CXHS1900040 | NCT02586350 | Head and neck cancer | Randomized | Parallel | Double-blinded | Ⅱb | PFS | 20.67 ms vs11.07 ms | 0.53(0.30- | MCCT | S(3) |
|  |  |  |  |  |  |  |  |  |  | 0.95) |  |  |
| Orelabrutinib | CXHS1900035 | NCT03493217 | Haematological Malignancies | Non-randomized | Singe-arm | Open | Ⅱ | ORR | 88.80% |  | MCCT |  |
| Orelabrutinib | CXHS2000008 | NCT03494179 | Haematological Malignancies | Non-randomized | Singe-arm | Open | Ⅱ | ORR | 76.70% |  | MCCT |  |
| Olaparib | JXHS1700046 | NCT01874353^c^ | Gynaecological Malignancies | Randomized | Parallel | Double-blinded | Ⅲ | PFS | 19.1ms vs.5.5ms | 0.3(0.22-0.41) | IMCT | Y(4) |
| Olaparib | JXHS1800061 | NCT01844986^c^ | Gynaecological Malignancies | Randomized | Parallel | Double-blinded | Ⅲ | CR | 56ms vs.13.8ms | 0.33(0.25-0.43) | IMCT | Y(4) |
| Olaparib | JXHS2100001 | NCT02987543^c^ | Genitourinary Cancers | Randomized | Parallel | Open | Ⅲ | OS | 14.7ms vs.14.3ms | 0.69(0.50-0.97) | IMCT | N(3) |
| Olverembatinib | CXHS2000038 | NCT04126681 | Haematological Malignancies | Non-randomized | Singe-arm | Open | Ⅱ | MaHR | 75.60% |  | MCCT |  |
| Obinutuzumab | JXSS1900059 | NCT01332968^c^ | Haematological Malignancies | Randomized | Parallel | Open | Ⅲ | PFS |  |  | IMCT |  |
| Osimertinib | JXHS1700005 | NCT02442349^c^ | Thoracic Malignancies | Non-randomized | Singe-arm | Open | Ⅱ | ORR | 60.20% |  | IMCT |  |
| Osimertinib | JXHS1700005 | NCT01802632 | Thoracic Malignancies | Non-randomized | Singe-arm | Open | Ⅱ | ORR | 66.10% |  | IMCT | S(4) |
| Osimertinib | JXHS1800054 | NCT02296125^c^ | Thoracic Malignancies | Randomized | Parallel | Double-blinded | Ⅲ | PFS | 18.9ms vs.10.2ms | 0.46(0.37-0.57) | IMCT | Y(4) |
| Osimertinib | JXHS2000151 | NCT02511106^c^ | Thoracic Malignancies | Randomized | Parallel | Double-blinded | Ⅲ | DFS | 65.8msvs. 21.9ms | 0.23(0.18-0.30) | IMCT | Y(A) |
| Bevacizumab | CXSS1800017 | NCT03169335 | Thoracic Malignancies | Randomized | Parallel | Double-blinded | Ⅲ | ORR | 52.26% |  | MCCT | S(2) |
| Bevacizumab | CXSS1900004 | NA/CTR20160848 | Thoracic Malignancies | Randomized | Parallel | Double-blinded | Ⅲ | ORR | 46.40% |  | MCCT | S(2) |
| Bevacizumab | CXSS2100008 | NCT03794440 | Gastrointestinal Cancers | Randomized | Parallel | Open | Ⅱ and Ⅲ | PFS | 4.6ms vs.2.8ms | 0.565(0.455- 0.701) | MCCT | N(3) |
| Blinatumomab | JXSS1900060 | NCT02013167 | Haematological Malignancies | Randomized | Parallel | Double-blinded | Ⅲ | PFS | 7.7ms vs.4.0ms | 0.71 (0.55-0.93) | IMCT |  |
| Blinatumomab | JXSS1900060 | NA/CTR20170176^b^ | Haematological Malignancies | Non-randomized | Singe-arm | Open | Ⅲ | CR | 47.80% |  | MCCT |  |
| Pyrotinib | CXHS1700013 | NCT02422199 | Breast Cancer | Randomized | Parallel | Open | Ⅱ | ORR | 78.50% |  | MCCT | N(3) |
| Dalpiciclib | CXHS2101006 | NCT03927456 | Breast Cancer | Randomized | Parallel | Double-blinded | Ⅲ | PFS | 15.7ms vs.7.2 ms | 0.424(0.309-0.581) | MCCT | N(3) |
| Dacomitinib | JXHS1800023 | NCT01774721^c^ | Thoracic Malignancies | Randomized | Parallel | Double-blinded | Ⅲ | PFS | 14.7 ms vs.9.2ms | 0.589(0.469-0.739) | IMCT | N(3) |
| Dacomitinib | JXHS1900092 | NCT01682083 | Skin Cancers | Randomized | Parallel | Double-blinded | Ⅲ | RFS | 52% vs.36% | 0.49(0.39-0.58) | IMCT | Y(4) |
| Daratumumab | JXSS1800023 | NCT02195479 | Haematological Malignancies | Randomized | Parallel | Open | Ⅲ | PFS | NE vs. 18.1ms | 0.50(0.38-0.65) | IMCT |  |
| Darolutamide | JXHS2000007 | NCT02200614^c^ | Genitourinary Cancers | Randomized | Parallel | Double-blinded | Ⅲ | MFS | 40.37 ms vs. 18.43 ms | 0.413(0.341-0.500) | IMCT | N(3) |
| Dasatinib | JXHL0600105 | NCT00481247 | Haematological Malignancies | Randomized | Singe-arm | Double-blinded | Ⅲ | CR | 77% |  | IMCT |  |
| Dinutuximab | JXSS2000048 | NCT01704716 | Head and neck cancer | Randomized | Parallel | Open | Ⅲ | EFS | 55.40% |  | IMCT | N(3) |
| Donafenib | CXHS2000010 | NCT02645981 | Gastrointestinal Cancers | Non-randomized | Singe-arm | Open | Ⅱ and Ⅲ | OS | 12.0ms vs.10.1ms | 0.839(0.706-0.996) | MCCT | N(3) |
| Erlotinib | JXHL0500008 | NCT00036647 | Thoracic Malignancies | Randomized | Parallel | Double-blinded | Ⅲ | PFS | 3.0ms vs.2.1ms | 0.63 | IMCT | Y(4) |
| Trastuzumab | JXSS1900012 | NCT01772472^c^ | Breast Cancer | Randomized | Parallel | Open | Ⅲ | IDFS | 88.27% vs.77.02%(3 year) |  | IMCT | Y(A) |
| Ensartinib | CXHS1800045 | NCT03215693 | Thoracic Malignancies | Non-randomized | Singe-arm | Open | Ⅱ | ORR | 48.70% |  | MCCT | Y(4) |
| Envolizumab | CXSS2000060 | NCT03667170 | Gastrointestinal Cancers | Non-randomized | Singe-arm | Open | Ⅱ | ORR | 39.20% |  | MCCT | N(3) |
| Enzalutamide | JXHS1900163 | NCT02003924^c^ | Genitourinary Cancers | Randomized | Parallel | Double-blinded | Ⅲ | MFS | 36.6ms vs. 14.7ms | 0.292 (0.241-0.352) | IMCT | Y(4) |
| Fruquintinib | CXHS1700008 | NCT02314819 | Gastrointestinal Cancers | Randomized | Parallel | Double-blinded | Ⅲ | OS | 9.3 ms vs.6.6ms | 0.65(0.51-0.83) | MCCT | N(3) |
| Furmonertinib | CXHS1900039 | NCT03787992 | Thoracic Malignancies | Randomized | Singe-arm | Double-blinded | II | ORR | 74.10% |  | MCCT | N(3) |
| Flumatinib | CXHS1800016 | NCT02204644 | Haematological Malignancies | Randomized | Parallel | Open | Ⅲ | MCR | 33.67% vs.18.27% |  | MCCT |  |
| Fuzuloparib | CXHS1900033 | NCT03509636 | Gynaecological Malignancies | Non-randomized | Singe-arm | Open | Ⅰb | ORR | 69.90% |  | MCCT | N(3) |
| Fuzuloparib | CXHS2000046 | NCT03863860 | Gynaecological Malignancies | Randomized | Parallel | Double-blinded | Ⅲ | PFS | 12.9ms vs.5.5ms | 0.245(0.17-0.36) | MCCT | S(3) |
| Gilteritinib | JXHS2000033 | NCT02421939^c^ | Haematological Malignancies | Randomized | Parallel | Open | Ⅲ | OS | 9.3 ms vs.5.6ms | 0.637(0.490-0.830) | IMCT |  |
| Carfilzomib | JXHS1900150 | NA/CTR20160857 | Haematological Malignancies | Non-randomized | Singe-arm | Open | Ⅲ | PFS | 5.6ms |  | MCCT |  |
| Camrelizumab | CXSS1800009 | NCT03155425 | Haematological Malignancies | Non-randomized | Singe-arm | Open | Ⅱ | ORR | 80.30% |  | MCCT |  |
| Camrelizumab | CXSS1900023 | NCT03764293 | Haematological Malignancies | Randomized | Parallel | Open | Ⅱ | ORR | 15.50% |  | MCCT |  |
| Camrelizumab | CXSS1900034 | NCT03134872 | Thoracic Malignancies | Randomized | Parallel | Double-blinded | Ⅲ | PFS | 11.3 ms vs.8.3ms | 0.599(0.453-0.791) | MCCT | S(3) |
| Camrelizumab | CXSS1900035 | NCT03134872 | Thoracic Malignancies | Randomized | Parallel | Double-blinded | Ⅲ | PFS | 8.3ms vs.6.2ms | 0.705(0.572-0.869) | MCCT | S(1) |
| Camrelizumab | CXSS2000045 | NCT03558191 | Head and neck cancer | Randomized | Singe-arm | Open | Ⅱ | ORR | 28.30% |  | MCCT | Y(3) |
| Cladribine | X0403028 | NCT00213135 | Haematological Malignancies | Randomized | Parallel | Double-blinded | Ⅲ | ARR | 0.14 vs. 0.33 | 0.67(0.48-0.93) | IMCT |  |
| Crizotinib | JXHL1300162 | NCT00932893^c^ | Thoracic Malignancies | Randomized | Parallel | Open | Ⅲ | PFS | 7.7ms vs.3.0 ms | 0.49 | IMCT | Y(4) |
| Lenalidomide | CXHS1400266 | NCT00480363 | Haematological Malignancies | Randomized | Singe-arm | Open | Ⅲ | CR+PR | 88.71% |  | SCCT |  |
| Lenalidomide | JXHS1600024 | NCT00689936^c^ | Haematological Malignancies | Non-randomized | Parallel | Open | Ⅲ | PFS | 25.5ms vs.21.2ms | 0.72 (0.61-0.85) | IMCT |  |
| Lenalidomide | JXHS1900154 | NCT01938001 | Haematological Malignancies | Randomized | Parallel | Double-blinded | Ⅲ | PFS | 39.4 ms vs.14.1ms | 0.46(0.34-0.62) | IMCT |  |
| Rituximab | CXSS1900030 | NA/CTR20160493 | Haematological Malignancies | Randomized | Parallel | Double-blinded | Ⅲ | ORR | 89.50% |  | SCCT |  |
| Lenvatinib | JXHS1700042 | NCT01761266^c^ | Gastrointestinal Cancers | Randomized | Parallel | Open | Ⅲ | OS | 13.6ms vs.12.3ms | 0.92(0.79-1.06) | IMCT | N(3) |
| Mitoxantrone | CXHS2000023 | NCT03776279 | Haematological Malignancies | Randomized | Singe-arm | Open | Ⅱ | ORR | 41.70% | DOR11.5ms | MCCT |  |
| Nivolumab | JXSS1700015 | NCT02613507^c^ | Thoracic Malignancies | Randomized | Parallel | Open | Ⅲ | OS | 11.9ms vs.9.5ms | 0.75(0.61-0.93) | IMCT | Y(5) |
| Nivolumab | JXSS1900001 | NCT02105636 | Head and neck cancer | Randomized | Parallel | Open | Ⅲ | OS | 7.7ms vs. 5.1ms | 0.68(0.54–0.86) | IMCT | N(3) |
| Nivolumab | JXSS1900037 | NCT02267343 | Gastrointestinal Cancers | Randomized | Parallel | Open | Ⅲ | OS | 5.3 msvs. 4.1 ms | 0.62(0.50–0.75) | IMCT | S(1) |
| Nivolumab | JXSS2000053 | NCT02899299 | Thoracic Malignancies | Randomized | Parallel | Open | Ⅲ | OS | 18.1 ms vs.14.1 ms | 0.73(0.61-0.87) | IMCT | N(3) |
| Niraparib | CXHS2000009 | NCT02655016 | Gynaecological Malignancies | Randomized | Parallel | Double-blinded | Ⅲ | PFS | 13.8ms vs.8.2ms | 0.62(0.502-0.755) | IMCT | N(3) |
| Pembrolizumab | JXSS1800002 | NCT01704287 | Skin Cancers | Randomized | Parallel | Double-blinded | Ⅱ | PFS | 2.9ms vs. 2.7ms | 0.57(0.45-0.73) | IMCT | Y(3) |
| Pembrolizumab | JXSS1800002 | NCT02821000^c^ | Skin Cancers | Non-randomized | Singe-arm | Open | 1Ⅰ | ORR | 16.70% | DOR8.4ms | MCCT |  |
| Pembrolizumab | JXSS2000010 | NCT02358031^c^ | Head and neck cancer | Randomized | Parallel | Double-blinded | Ⅲ | OS | 13.0 ms vs. 10.7 ms | 0.77(0.63-0.93) | IMCT | N(3) |
| Pembrolizumab | JXSS2000063 | NCT02563002 | Gastrointestinal Cancers | Randomized | Parallel | Open | Ⅲ | OS | 16.5 ms vs.8.2ms | 0.60(0.45-0.80) | IMCT | Y(4) |
| Pamiparib | CXHS2000021 | NCT03333915 | Gynaecological Malignancies | Non-randomized | Singe-arm | Open | Ⅰ and Ⅱ | ORR | 69.30% |  | MCCT | N(3) |
| Pertuzumab | JXSS1800020 | NCT02586025^c^ | Breast Cancer | Randomized | Parallel | Double-blinded | Ⅲ | CR | 39.3%vs.21.8% |  | IMCT | Y(A) |
| Palbociclib | JXHL1500268 | NCT00721409^b^ | Breast Cancer | Randomized | Parallel | Open | Ⅰ | ORR | 19.20% |  | IMCT | S(1) |
| Palbociclib | JXHL1500268 | NCT01740427 | Breast Cancer | Randomized | Parallel | Double-blinded | Ⅲ | PFS | 24.8ms vs.14.5ms | 0.576(0.46-0.72) | IMCT | N(3) |
| Pemetrexed | J0401653 | NA | Thoracic Malignancies | Randomized | Parallel | Open | Ⅲ | OS | 12.1ms vs.9.3ms |  | IMCT | S(3) |
| Bortezomib | JYHB0500445 | NCT00048230^c^ | Haematological Malignancies | Randomized | Singe-arm | Open | Ⅲ | OS | 56.4ms vs.43.1ms | 0.695 | IMCT |  |
| Bortezomib | JYHB0500445 | NA^b^ | Haematological Malignancies | Randomized | Singe-arm | Open | Ⅱ | ORR | 27.70% |  | IMCT |  |
| Pralatrexate | JXHS1800080 | NCT00364923 | Haematological Malignancies | Non-randomized | Singe-arm | Open | Ⅱ | ORR | 27% |  | IMCT |  |
| Pralatrexate | JXHS1800080 | NCT03349333 | Haematological Malignancies | Non-randomized | Singe-arm | Open | Ⅲ | ORR | 52.1%% |  | MCCT |  |
| Pralsetinib | JXHS2000131 | NCT03037385^c^ | Thoracic Malignancies | Non-randomized | Singe-arm | Open | Ⅰ and Ⅱ | ORR | 59% |  | IMCT | N(3) |
| Trametinib | JXHS1900091 | NCT01682083 | Thoracic Malignancies | Randomized | Parallel | Open | Ⅲ | RFS | 93.1ms vs.16.6ms | 0.47 0.39-0.58) | IMCT | Y(4) |
| Trastuzumab | CXSS1900021 | NCT03084237^c^ | Breast Cancer | Randomized | Parallel | Double-blinded | Ⅲ | ORR | 71.30% |  | IMCT | S(3) |
| Regorafenib | JXHS1500103 | NCT01103323 | Gastrointestinal Cancers | Randomized | Parallel | Double-blinded | Ⅱ | OS | 10.6 ms vs. 7.8 ms | 0.774(0.636- | IMCT | N(4) |
|  |  |  |  |  |  |  |  |  |  | 0.942 |  |  |
| Regorafenib | JXHS1500103 | NCT01584830^c^ | Gastrointestinal Cancers | Randomized | Parallel | Double-blinded | Ⅲ | OS | 8.8 ms vs. 6.3 ms | 0.550(0.395- | IMCT |  |
|  |  |  |  |  |  |  |  |  |  | 0.765) |  |  |
| Relmacabtagene Autoleucel | CXSS2000036 | NCT04089215^b^ | Haematological Malignancies | Non-randomized | Singe-arm | Open | Ⅱ | ORR | 58.30% |  | MCCT |  |
| Ripretinib | JXHS2000121 | NCT03353753 | Gastrointestinal Cancers | Randomized | Parallel | Open | Ⅲ | PFS | 6.3ms vs.1.0ms | 0.15(0.09-0.25) | IMCT | N(3) |
| Ripretinib | JXHS2000121 | NCT04282980^b^ | Gastrointestinal Cancers | Randomized | Parallel | Open | Ⅱ | PFS | 7.2ms vs.1.9ms |  | MCCT |  |
| Ceritinib | JXHS1700053 | NCT01828099 | Thoracic Malignancies | Randomized | Parallel | Open | Ⅲ | PFS | 5.4ms vs.1.6 ms | 0.49(0.36-0.67) | IMCT | Y(4) |
| Ceritinib | JXHS1700053 | NA^b^ | Thoracic Malignancies | Non-randomized | Singe-arm | Open | Ⅰ and Ⅱ | ORR | 40.80% | DOR8.5ms | MCCT |  |
| Chidamide | CXHS2000012 | NA/CTR20211151 | Thoracic Malignancies | Non-randomized | Singe-arm | Open | Ⅱ | ORR | 42.90% |  | MCCT | N(2) |
| Sunitinib | JXHL0500229 | NCT00083889 | Genitourinary Cancers | Randomized | Parallel | Open | Ⅲ | OS | 26.4ms vs. 21.8ms | 0.82(0.67-1) | IMCT | Y(4) |
| Sulfatinib | CXHS1900034 | NCT02588170 | Gastrointestinal Cancers | Randomized | Parallel | Double-blinded | Ⅲ | PFS | 9.2ms vs.3.8ms | 0.334(0.223-0.499) | MCCT | Y(3) |
| Sulfatinib | CXHS1900034 | NA | Gastrointestinal Cancers | Non-randomized | Singe-arm | Open | Ⅱ | ORR | 17.30% |  | MCCT | N(3) |
| Sorafenib | JXHL0500210 | NCT00105443 | Genitourinary Cancers | Randomized | Parallel | Double-blinded | Ⅲ | OS | 10.7ms vs.7.9ms | 0.69(0.55-0.87) | IMCT | N(3) |
| Sorafenib | JXHS1600040 | NCT00984282^c^ | Endocrine Tumours | Randomized | Parallel | Double-blinded | Ⅲ | PFS | 10.8ms vs.5.8ms | 0.59(0.45-0.76) | IMCT | Y(2) |
| Toripalimab | CXSS1800006 | NCT04180995 | Skin Cancers | Non-randomized | Singe-arm | Open | Ⅱ | ORR | 17.30% |  | MCCT | N(2) |
| Toripalimab | CXSS2000016 | NCT02915432 | Head and neck cancer | Non-randomized | Singe-arm | Open | Ⅱ | ORR | 23.90% | DOR14.9ms | MCCT | S(3) |
| Toripalimab | CXSS2000018 | NCT03113266 | Genitourinary Cancers | Non-randomized | Singe-arm | Open | Ⅱ | ORR | 27.20% | DOR19.7ms | MCCT | S(3) |
| Tislelizumab | CXSS1800019 | NCT03209973 | Haematological Malignancies | Non-randomized | Singe-arm | Open | Ⅱ | ORR | 76.90% |  | MCCT |  |
| Tislelizumab | CXSS1900025 | NCT04004221^c^ | Genitourinary Cancers | Non-randomized | Singe-arm | Open | Ⅱ | ORR | 24.80% | DOR not yet reached，PFS2ms | IMCT | S(3) |
| Tislelizumab | CXSS2000033 | NCT03358875^c^ | Gastrointestinal Cancers | Non-randomized | Singe-arm | Open | Ⅱ | ORR | 13.30% |  | IMCT | N(4) |
| Tislelizumab | CXSS2101004 | NCT03736889 | Solid cancer | Non-randomized | Singe-arm | Open | Ⅱ | ORR | 41.20% |  | MCCT | S(3) |
| Brentuximab | JXSS1900015 | NCT00848926 | Haematological Malignancies | Non-randomized | Singe-arm | Open | Ⅱ | ORR | 75% |  | IMCT |  |
| Brentuximab | JXSS1900015 | NA^b^ | Haematological Malignancies | Non-randomized | Singe-arm | Open | Ⅱ | ORR | 50% |  | MCCT |  |
| Disitamab vedotin | CXSS2000044 | NCT03556345 | Gastrointestinal Cancers | Non-randomized | Singe-arm | Open | Ⅱ | ORR | 23.60% |  | MCCT | N(1) |
| Disitamab vedotin | CXSS2101011 | NCT03809013 | Genitourinary Cancers | Non-randomized | Singe-arm | Open | Ⅱ | ORR | 50.00% | DOR8.3ms | MCCT |  |
| Vemurafenib | JXHS1600039 | NA/CTR20130184^b^ | Skin Cancers | Non-randomized | Singe-arm | Open | Ⅰ | CR+PR | 52% | DOR9.1ms | MCCT |  |
| Vemurafenib | JXHS1600039 | NCT01006980 | Skin Cancers | Randomized | Parallel | Open | Ⅲ | OS | 6.2ms vs.4.5ms | 0.44(0.33-0.59) | IMCT | Y(4) |
| Chidamide | CXHS1300047 | NA | Haematological Malignancies | Non-randomized | Singe-arm | Open | Ⅱ | ORR | 28% |  | MCCT |  |
| Chidamide | CXHS1800033 | NCT02482753 | Breast Cancer | Randomized | Parallel | Double-blinded | Ⅲ | PFS | 9.2 ms vs.3.8 ms | 0.75 (0.58-0.98) | MCCT | S(2) |
| Cetuximab | J0402422 | NCT00154102 | Gastrointestinal Cancers | Randomized | Parallel | Open | Ⅲ | OS | 28.4ms vs.20.2ms | 0.84 | IMCT | N(4) |
| Cetuximab | JXSS1900014 | NCT02383966^b^ | Head and neck cancer | Randomized | Parallel | Open | Ⅲ | PFS | 5.5 ms vs.4.2ms | 0.566(0.400-0.803) | MCCT |  |
| Cetuximab | JXSS1900014 | NCT00122460 | Head and neck cancer | Randomized | Parallel | Open | Ⅲ | OS | 10.1 ms vs.7.4ms | 0.80(0.64- | IMCT | N(3) |
|  |  |  |  |  |  |  |  |  |  | 0.99) |  |  |
| Sintilimab | CXSS1800008 | NCT03114683 | Haematological Malignancies | Randomized | Singe-arm | Open | Ⅱ | ORR | 85.30% |  | MCCT |  |
| Sintilimab | CXSS2100009 | NCT03794440 | Gastrointestinal Cancers | Randomized | Parallel | Open | Ⅲ | PFS | 4.6ms vs.2.8ms | 0.565(0.455-0.701) | MCCT | S(3) |
| Ibrutinib | JXHS1600066 | NCT01236391 | Haematological Malignancies | Non-randomized | Singe-arm | Open | Ⅱ | ORR | 68.50% |  | IMCT |  |
| Ibrutinib | JYHB1800207 | NCT02165397 | Haematological Malignancies | Randomized | Parallel | Double-blinded | Ⅲ | PFS | 14.6ms vs.6.2ms | 0.43(0.32-0.58) | MCCT |  |
| Inetetamab | CXSS1800023 | NA/CTR20160389 | Breast Cancer | Randomized | Parallel | Double-blinded | Ⅲ | PFS | 39.1ms vs.14ms | 0.24(0.16-0.36) | MCCT | N(3) |
| Ipilimumab | JXSS2000055 | NCT02899299 | Thoracic Malignancies | Randomized | Parallel | Open | Ⅲ | OS | 18.1ms vs.14.1ms | 0.74(0.6-0.91) | IMCT | N(3) |
| Ixazomib | JXHL1600072 | NCT01564537 | Haematological Malignancies | Randomized | Parallel | Double-blinded | Ⅲ | PFS | 20.6ms vs.14.7ms | 0.82(0.67-1) | IMCT |  |
| Inotuzumab ozogamicin | JXSS2000001 | NCT01564784^c^ | Haematological Malignancies | Randomized | Parallel | Open | Ⅲ | CR | 80.70% |  | IMCT |  |
| Utidelone | CXHS1800005 | NCT02253459 | Breast Cancer | Randomized | Parallel | Open | Ⅲ | PFS | 8.44ms vs.4.14ms | 0.47(0.37,0.59) | MCCT | N(2) |
| Zanubrutinib | CXHS1800024 | NCT03206970 | Haematological Malignancies | Non-randomized | Singe-arm | Open | Ⅱ | ORR | 83.70% |  | MCCT |  |
| Zanubrutinib | CXHS1800030 | NCT03332173 | Haematological Malignancies | Non-randomized | Singe-arm | Open | Ⅱ | ORR | 62.60% |  | MCCT |  |
| Zanubrutinib | CXHS2000037 | NCT03846427 | Haematological Malignancies | Randomized | Singe-arm | Open | Ⅱ | ORR | 69.80% |  | MCCT |  |
| Decitabine | JXHL0700089 | NCT00043381 | Haematological Malignancies | Randomized | Parallel | Open | Ⅲ | ORR | 25% |  | MCCT |  |
| Abivertinib | CXHS1800008 | NCT03452592 | Thoracic Malignancies | Non-randomized | Singe-arm | Open | Ⅱ | ORR | Not approved |  | MCCT |  |
| Dicycloplatin | CXHS0900153 | NA/CTR20170903 | Solid cancer | Non-randomized | Singe-arm | Open | Ⅱ | PSA | Not approved |  | MCCT |  |
| Geptanolimab | CXSS2000042 | NA/CTR20180442 | Haematological Malignancies | Non-randomized | Singe-arm | Open | Ⅱ | ORR | Not approved |  | MCCT |  |

Note: **IMCT**: International Multicenter Clinical Trial. **CJMRCT**: China Joining Multi-Regional Clinical Trials. **MCCT:** Multi-Center Clinical Trial. **SCCT**: Single Center Clinical Trial.

**OS:** Overall Survival. **PFS:** Progression-Free Survival. Overall Response Rate (**ORR**) includes Partial Response (**PR**) and Complete Response (**CR**). **MFS:** Metastasis- free survival.

**DFS:** Disease free survival. **EFS:** Event-free survival.

## eTable S6: Study characteristics of generic drug approved by EP from 2005-2021

| **Genetic name** | **Handling No.** | T**rial objective** | **Randomization** | **Design type** | **Degree of blindness** | **Trials Phase** | **The number of enrollments** | **Evaluation indicators** |
| --- | --- | --- | --- | --- | --- | --- | --- | --- |
| Anastrozole | CYHS1800473 | Bioequivalence | Randomized | Crossover | Open | Ⅰ | 48 | AUC |
| Anastrozole | CYHS1900269 | Bioequivalence | Randomized | Crossover | Open | Ⅰ | 46 | AUC |
| Azacitidine | CYHS1600206 | Bioequivalence | Randomized | Crossover | Open | Ⅰ | 46 | AUC |
| Bendamustine | CYHS1900557 | Bioequivalence | Randomized | Crossover | Open | Ⅰ | 10 | AUC |
| Docetaxel | CYHS1700558 | Bioequivalence | Randomized | Crossover | Open | Ⅰ |  | AUC |
| Erlotinib | CYHS1790011 | Bioequivalence | Randomized | Crossover | Open | Ⅰ | 42 | AUC |
| Fulvestrant | CYHS1900083 | Bioequivalence | Randomized | Crossover | Open | Ⅰ | 42 | DLT, MTD |
| Gefitinib | CYHS1490010 | Bioequivalence | Randomized | crossover | Open | Ⅰ | 59 | AUC |
| Letrozole | CYHS1900655 | Bioequivalence | Randomized | crossover | Open | Ⅰ | 48 | AUC |
| Pemetrexed | CYHS1600043 | Bioequivalence | Randomized | Crossover | Open | Ⅰ |  | AUC |
| Pemetrexed | CYHS1800165 | Bioequivalence | Randomized | Crossover | Open | Ⅰ |  | AUC |
| Pemetrexed | CYHS1900295 | Bioequivalence | Randomized | Crossover | Open | Ⅰ |  | AUC |
| Bortezomib | CYHS1900843 | Bioequivalence | Randomized | Crossover | Open | Ⅰ |  | AUC |
| Sorafenib | CYHS1900700 | Bioequivalence | Randomized | Crossover | Open | Ⅰ | 42 | AUC |
| Temozolomide | CYHS1700055 | Bioequivalence | Randomized | Crossover | Open | Ⅰ | 28 | AUC |
| Imatinib | CYHS1700601 | Bioequivalence | Randomized | Crossover | Open | Ⅰ | 24 | AUC |
| Paclitaxel(Albumin Bound) | CYHS1600152 | Bioequivalence | Randomized | Crossover | Open | Ⅰ | 40 | AUC |
| Paclitaxel(Albumin Bound) | CYHS1790004 | Bioequivalence | Randomized | Crossover | Open | Ⅰ | 24 | AUC |
| Paclitaxel | CYHS1900682 | Bioequivalence | Randomized | Crossover | Open | Ⅰ | 48 | AUC |
| Calcium Levofolinate | CXHS1700029 | Bioequivalence | Randomized | Crossover | Open | Ⅰ |  | AUC |

Note: **DLT:** Dose-Limiting Toxicity. **MTD:** Maximum Tolerated Dose. **AUC:** Area Under Curve.

## eTable S7: Number of patients enrolled in 137 pivotal trials supporting oncology indications approved under the EA program (2005–2021)

| **Genetic name** | **Handling No.** | **Enrollments No.** | **Genetic name** | **Handling No.** | **Enrollments No.** | **Genetic name** | **Handling No.** | **Enrollments No.** |
| --- | --- | --- | --- | --- | --- | --- | --- | --- |
| Abemaciclib | JXHS1900144 | 493 | Donafenib | CXHS2000010 | 668 | Regorafenib | JXHS1500103 | 760 |
| Abemaciclib | JXHS1900144 | 306 | Erlotinib | JXHL0500008 | 163 | Regorafenib | JXHS1500103 | 204 |
| Abiraterone | JXHS1800011 | 1199 | Trastuzumab | JXSS1900012 | 1487 | Relmacabtagene Autoleucel | CXSS2000036 | 59 |
| Avapritinib | JXHS2000050 | 43 | Ensartinib | CXHS1800045 | 160 | Ripretinib | JXHS2000121 | 129 |
| Avapritinib | JXHS2000050 | 50 | Envolizumab | CXSS2000060 | 103 | Ripretinib | JXHS2000121 | 39 |
| Afatinib | JXHS1600009 | 364 | Enzalutamide | JXHS1900163 | 1401 | Ceritinib | JXHS1700053 | 231 |
| Axicabtagene ciloleucel | CXSS2000006 | 101 | Fruquintinib | CXHS1700008 | 416 | Ceritinib | JXHS1700053 | 103 |
| Axicabtagene ciloleucel | CXSS2000006 | 27 | Furmonertinib | CXHS1900039 | 220 | Chidamide | CXHS2000012 | 70 |
| Alectinib | JXHS1800001 | 303 | Flumatinib | CXHS1800016 | 393 | Sunitinib | JXHL0500229 | 750 |
| Almonertinib | CXHS1900011 | 244 | Fuzuloparib | CXHS1900033 | 113 | Sulfatinib | CXHS1900034 | 219 |
| Almonertinib | CXHS2101017 | 429 | Fuzuloparib | CXHS2000046 | 252 | Sulfatinib | CXHS1900034 | 81 |
| Apalutamide | JXHS1900036 | 1207 | Gilteritinib | JXHS2000033 | 371 | Sorafenib | JXHL0500210 | 602 |
| Apalutamide | JXHS1900156 | 1052 | Carfilzomib | JXHS1900150 | 126 | Sorafenib | JXHS1600040 | 417 |
| Apatinib | CXHS1100133 | 144 | Camrelizumab | CXSS1800009 | 75 | Toripalimab | CXSS1800006 | 128 |
| Atezolizumab | JXSS2000002 | 501 | Camrelizumab | CXSS1900023 | 220 | Toripalimab | CXSS2000016 | 190 |
| Atezolizumab | JXSS2000033 | 572 | Camrelizumab | CXSS1900034 | 412 | Toripalimab | CXSS2000018 | 151 |
| Axitinib | JXHS1400080 | 723 | Camrelizumab | CXSS1900035 | 448 | Tislelizumab | CXSS1800019 | 70 |
| Icotinib | CXHL0502177 | 134 | Camrelizumab | CXSS2000045 | 156 | Tislelizumab | CXSS1900025 | 113 |
| Icotinib | CXHS2000030 | 283 | Cladribine | X0403028 | 1326 | Tislelizumab | CXSS2000033 | 249 |
| Anlotinib | CXHS1700003 | 437 | Crizotinib | JXHL1300162 | 347 | Tislelizumab | CXSS2101004 | 80 |
| Anlotinib | CXHS1800025 | 143 | Lenalidomide | CXHS1400266 | 124 | Brentuximab | JXSS1900015 | 102 |
| Anlotinib | CXHS1900040 | 91 | Lenalidomide | JXHS1600024 | 1623 | Brentuximab | JXSS1900015 | 58 |
| Orelabrutinib | CXHS1900035 | 80 | Lenalidomide | JXHS1900154 | 358 | Brentuximab | JXSS1900015 | 60 |
| Orelabrutinib | CXHS2000008 | 86 | Rituximab | CXSS1900030 | 420 | Disitamab vedotin | CXSS2000044 | 127 |
| Olaparib | JXHS1700046 | 295 | Lenvatinib | JXHS1700042 | 954 | Disitamab vedotin | CXSS2101011 | 43 |
| Olaparib | JXHS1800061 | 391 | Mitoxantrone | CXHS2000023 | 108 | Vemurafenib | JXHS1600039 | 46 |
| Olaparib | JXHS2100001 | 442 | Nivolumab | JXSS1700015 | 504 | Chidamide | CXHS1300047 | 83 |
| Olverembatinib | CXHS2000038 | 41 | Nivolumab | JXSS1900001 | 361 | Chidamide | CXHS1800033 | 348 |
| Osimertinib | JXHS1700005 | 171 | Nivolumab | JXSS1900037 | 493 | Cetuximab | J0402422 | 1221 |
| Osimertinib | JXHS1700005 | 201 | Nivolumab | JXSS2000053 | 605 | Cetuximab | JXSS1900014 | 243 |
| Osimertinib | JXHS1800054 | 556 | Niraparib | CXHS2000009 | 733 | Cetuximab | JXSS1900014 | 442 |
| Osimertinib | JXHS2000151 | 682 | Pembrolizumab | JXSS1800002 | 540 | Sintilimab | CXSS1800008 | 136 |
| Bevacizumab | CXSS1800017 | 535 | Pembrolizumab | JXSS1800002 | 103 | Sintilimab | CXSS2100009 | 595 |
| Bevacizumab | CXSS1900004 | 450 | Pembrolizumab | JXSS2000010 | 882 | Ibrutinib | JXHS1600066 | 111 |
| Bevacizumab | CXSS2100008 | 571 | Pembrolizumab | JXSS2000063 | 307 | Ibrutinib | JYHB1800207 | 150 |
| Blinatumomab | JXSS1900060 | 405 | Pamiparib | CXHS2000021 | 113 | Inetetamab | CXSS1800023 | 341 |
| Blinatumomab | JXSS1900060 | 67 | Pertuzumab | JXSS1800020 | 808 | Ipilimumab | JXSS2000055 | 303 |
| Pyrotinib | CXHS1700013 | 128 | Palbociclib | JXHL1500268 | 26 | Ixazomib | JXHL1600072 | 722 |
| Dalpiciclib | CXHS2101006 | 361 | Palbociclib | JXHL1700152 | 666 | Ixazomib | JXHL1600072 | 115 |
| Dacomitinib | JXHS1800023 | 452 | Pemetrexed | J0401653 | 448 | Inotuzumab ozogamicin | JXSS2000001 | 326 |
| Dacomitinib | JXHS1900092 | 870 | Bortezomib | JYHB0500445 | 487 | Utidelone | CXHS1800005 | 405 |
| Daratumumab | JXSS1800023 | 706 | Bortezomib | JYHB0500445 | 202 | Zanubrutinib | CXHS1800024 | 86 |
| Daratumumab | JXSS1800023 | 106 | Pralatrexate | JXHS1800080 | 115 | Zanubrutinib | CXHS1800030 | 91 |
| Darolutamide | JXHS2000007 | 1509 | Pralsetinib | JXHS2000131 | 589 | Zanubrutinib | CXHS2000037 | 44 |
| Dasatinib | JXHL0600105 | 547 | Trametinib | JXHS1900091 | 432 | Decitabine | JXHL0700089 | 170 |
| Dinutuximab | JXSS2000048 | 367 | Trastuzumab | CXSS1900021 | 649 |  |  |  |

* The median number of enrollments was 303.Q1 was 113, and Q3 was 501.

## eFigure 1: Number of patients enrolled in 137 pivotal trials supporting oncology indications approved under the EA program (2005–2021)


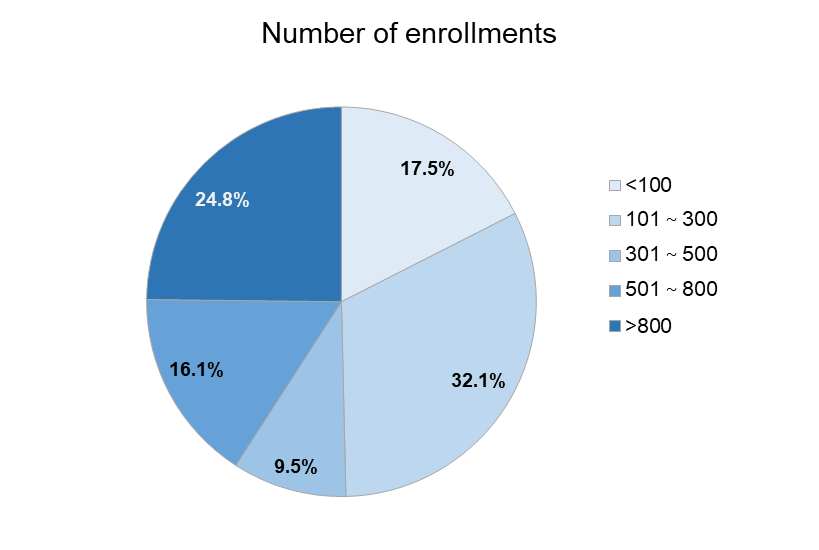

Supplement: Supplementary file 1 — Supplementary Material 1. [file 12885_2026_15749_MOESM1_ESM.docx]
