# Supplementary material for: Accelerating cancer therapy review: a cross-sectional analysis of expedited approval in China, 2005–2021
Source: BMC Cancer. 2026 Feb 16;26:390. doi: 10.1186/s12885-026-15749-1 (PMC13015170; doi:10.1186/s12885-026-15749-1)
Supplement: Supplementary file 2 — Supplementary Material 2. [file 12885_2026_15749_MOESM2_ESM.docx]

STROBE Checklist

**Article title:** Accelerating Cancer Therapy Review: A Cross-Sectional Analysis of Expedited Approval in China, 2005–2021

**Study design:** Cross-sectional study

STROBE Statement—Checklist of items that should be included in reports of ***cross-sectional studies***

| Section | Item No. | Recommendation | Page |
| --- | --- | --- | --- |
| **Title and abstract** | 1(a) | Indicate the study’s design with a commonly used term in the title or the abstract | Page 1 |
|  | 1(b) | Provide in the abstract an informative and balanced summary of what was done and what was found | Page 1 |
| **Introduction** | 2 | Explain the scientific background and rationale for the investigation being reported | Pages 2–3 |
|  | 3 | State specific objectives, including any prespecified hypotheses | Page 3 |
| **Methods** | 4 | Present key elements of study design early in the paper | Page 3 |
|  | 5 | Describe the setting, locations, and relevant dates, including periods of data collection | Pages 3-4 |
|  | 6(a) | Give the eligibility criteria and the sources and methods of selection | Pages 3-4 |
|  | 7 | Clearly define all outcomes, exposures, predictors, and effect modifiers | Pages 3-4 |
|  | 8* | Give sources of data and details of methods of assessment | Pages 3-4 |
|  | 9 | Describe any efforts to address potential sources of bias | Page 3-4 |
|  | 10 | Explain how the study size was arrived at | Pages 3-4 |
|  | 11 | Explain how quantitative variables were handled in the analyses | Page 3-4 |
|  | 12(a) | Describe all statistical methods | Pages 3-4 |
|  | 12(b) | Describe any methods used to examine subgroups and interactions | Page 3-4 |
|  | 12(c) | Explain how missing data were addressed | Page 3-4 |
|  | 12(d) | If applicable, describe analytical methods taking account of sampling strategy | Not applicable |
|  | 12(e) | Describe any sensitivity analyses | Not applicable |
| **Results** | 13(a) | Report numbers of observations at each stage of the study | Pages 4-6 |
|  | 13(b) | Give reasons for non-participation at each stage | Not applicable |
|  | 13(c) | Consider use of a flow diagram | Not applicable |
|  | 14(a) | Give characteristics of study subjects | Pages 7-10 |
|  | 14(b) | Indicate number of observations with missing data | Not applicable |
|  | 15 | Report numbers of outcome events or summary measures | Pages 7-10 |
|  | 16(a) | Give unadjusted estimates and, if applicable, adjusted estimates | Pages 7–10 |
|  | 16(b) | Report category boundaries when continuous variables were categorized | Page 7–10 |
|  | 16(c) | If relevant, translate relative risk into absolute risk | Not applicable |
|  | 17 | Report other analyses done (e.g., subgroup analyses) | Pages 7–10 |
| **Discussion** | 18 | Summarise key results with reference to study objectives | Pages 12-13 |
|  | 19 | Discuss limitations of the study | Page 16 |
|  | 20 | Give a cautious overall interpretation of results | Pages 13-16 |
|  | 21 | Discuss the generalisability of the study results | Pages 13-16 |
| **Other information** | 22 | Give the source of funding and the role of the funders | Page 18 |

*Give information separately for exposed and unexposed groups.

**Note:** An Explanation and Elaboration article discusses each checklist item and gives methodological background and published examples of transparent reporting. The STROBE checklist is best used in conjunction with this article (freely available on the Web sites of PLoS Medicine at http://www.plosmedicine.org/, Annals of Internal Medicine at http://www.annals.org/, and Epidemiology at http://www.epidem.com/). Information on the STROBE Initiative is available at www.strobe-statement.org.
